# Data-driven methods for dengue prediction and surveillance using real-world and Big Data: A systematic review

**DOI:** 10.1371/journal.pntd.0010056

**Published:** 2022-01-07

**Authors:** Emmanuelle Sylvestre, Clarisse Joachim, Elsa Cécilia-Joseph, Guillaume Bouzillé, Boris Campillo-Gimenez, Marc Cuggia, André Cabié

**Affiliations:** 1 Université de Rennes, CHU Rennes, INSERM, LTSI – UMR 1099, Rennes, France; 2 CHU Martinique, Centre de Données Cliniques, Martinique, France; 3 CHU Martinique, Pôle de Cancérologie Hématologie Urologie, Registre Général des Cancers de la Martinique, Martinique, France; 4 CHU Martinique, Pôle de Cancérologie Hématologie Urologie, Martinique Cancer Data Hub, Martinique, France; 5 Centre de Lutte Contre le Cancer Eugène Marquis, Rennes, France; 6 CHU Martinique, Infectious and Tropical Diseases Unit, Martinique, France; 7 CHU Martinique, INSERM, CIC-1424, Martinique, France; 8 PCCEI, Université de Montpellier, INSERM, EFS, Université Antilles, Montpellier, France; Universidade Federal de Alagoas - Campus Arapiraca, BRAZIL

## Abstract

**Background:**

Traditionally, dengue surveillance is based on case reporting to a central health agency. However, the delay between a case and its notification can limit the system responsiveness. Machine learning methods have been developed to reduce the reporting delays and to predict outbreaks, based on non-traditional and non-clinical data sources. The aim of this systematic review was to identify studies that used real-world data, *Big Data* and/or machine learning methods to monitor and predict dengue-related outcomes.

**Methodology/Principal findings:**

We performed a search in PubMed, Scopus, Web of Science and grey literature between January 1, 2000 and August 31, 2020. The review (ID: CRD42020172472) focused on data-driven studies. Reviews, randomized control trials and descriptive studies were not included. Among the 119 studies included, 67% were published between 2016 and 2020, and 39% used at least one novel data stream. The aim of the included studies was to predict a dengue-related outcome (55%), assess the validity of data sources for dengue surveillance (23%), or both (22%). Most studies (60%) used a machine learning approach. Studies on dengue prediction compared different prediction models, or identified significant predictors among several covariates in a model. The most significant predictors were rainfall (43%), temperature (41%), and humidity (25%). The two models with the highest performances were Neural Networks and Decision Trees (52%), followed by Support Vector Machine (17%). We cannot rule out a selection bias in our study because of our two main limitations: we did not include preprints and could not obtain the opinion of other international experts.

**Conclusions/Significance:**

Combining real-world data and *Big Data* with machine learning methods is a promising approach to improve dengue prediction and monitoring. Future studies should focus on how to better integrate all available data sources and methods to improve the response and dengue management by stakeholders.

## Author summary

Dengue is one of the most important arbovirus infections in the world and its public health, societal and economic burden is increasing. Although the majority of dengue cases are asymptomatic or mild, severe disease forms can lead to death. For this reason, early diagnosis and monitoring of dengue are crucial to decrease mortality. However, most endemic regions still rely on traditional monitoring methods, despite the growing availability of novel data sources and data-driven methods based on real-world data, *Big Data*, and machine learning algorithms. In this systematic review, we identified and analyzed studies that used these novel approaches for dengue monitoring and/or prediction. We found that novel data streams, such as Internet search engines and social media platforms, and machine learning methods can be successfully used to improve dengue management, but are still vastly ignored in real life. These approaches should be combined with traditional methods to help stakeholders better prepare for each outbreak and improve early responsiveness.

## Introduction

Dengue virus (DENV) is an arbovirus transmitted to humans by *Aedes aegypti* or *Aedes albopictus* female mosquitoes [1]. The incidence of dengue, the disease caused by DENV, has rapidly increased around the world in recent decades [2] due to population growth, urbanization, increased travel, and insufficient vector control [3]. The World Health Organization (WHO), considers dengue a major global public health challenge in the tropical and subtropical regions [4]. Today, dengue is one of the most important vector-borne diseases in the world and recent studies on its prevalence estimate that 3.9 billion people are at risk of transmission, with 390 million infections and 96 million symptomatic cases per year [1,5]. Although most infections are asymptomatic or are characterized by intense flu-like symptoms that last up to 10 days [6], severe forms of dengue hemorrhagic fever/dengue shock syndrome can also occur [7] and might lead to death. Mortality due to dengue can be greatly reduced by early diagnosis, appropriate clinical management [3,7].

Most dengue-endemic regions (mainly South-East Asia, the Americas, and the Pacific region) rely on traditional surveillance, based on hospital syndromic reporting and laboratory confirmation of a subset of cases to a central health agency [3,8]. The method is very accurate, but is hampered by its lack of responsiveness with substantial delays between a case and its notification [8], which can limit the health system ability/rapidity to put in place appropriate measures to avoid drastic consequences. Moreover, this traditional surveillance system is expensive, due to the time needed to aggregate and manually validate data [9]. These limitations have prompted researchers to investigate other solutions. Many studies have described alternative methods, such as mobile, digital and Internet-based systems, to efficiently crowd-source data from the community [3]. However, these approaches have not been translated yet into the standard dengue management practice. Yet, they are relevant for all dimensions of dengue management, such as monitoring, clinical management, and dengue outbreak forecasting [3,8]. Over the years, scientists have developed statistical and machine learning models to reduce the reporting delays and monitor new cases in almost real-time, but also to accurately use non-traditional and non-clinical data sources (e.g. Internet search engines and social media platforms) to predict communicable disease outbreaks [10–13], including dengue. Many studies have proposed new strategies based on *Big Data* and machine learning models to improve dengue outbreak management. However, recent systematic reviews only examined the relevance and usefulness of Internet-based surveillance systems in emerging tropical disease management [8,14], and they did not focus specifically on dengue management. Furthermore, recent systematic reviews on dengue analyzed monitoring [15], vaccine efficacy [16], epidemiological trends [17,18], the overall disease burden [19–21] and clinical prognosis models [22], but they did not discuss these new methods to improve dengue management.

Therefore, the first aim of this systematic review was to identify and describe all real-world and *Big Data*-based methods used to monitor and predict/forecast dengue-related outcomes, regardless of the region and/or population. The second aim was to analyze several features of these studies, such as the data sources and their origin, the different outcome types (e.g. epidemiological and clinical outcomes), the chosen statistical methods, and their performance and variability based on the population and location.

## Methods

This systematic review was performed following the “Preferred Reporting Items for Systematic Reviews and Meta-Analyses” (PRISMA) guidelines [23]. Four reviewers (ES, CJ, AC and MC) developed the systematic review protocol. The literature search was performed in September 2020. The study protocol was registered on the PROSPERO registry of systematic reviews (ID: CRD42020172472).

### Eligibility criteria

The review focused on studies that used real-world data, *Big Data* and/or machine learning methods to monitor, predict and/or forecast dengue outbreaks or dengue-related outcomes (clinical or epidemiological). Studies from any country (also regions outside endemic regions) were included, without any language filter. Analyses could be performed on past or future data.

### Inclusion criteria

- Dengue diagnosis based on the standard WHO definition [7] valid at the time of the study
- Studies on humans, regardless of age, sex, and disease severity
- Studies using real-world data (including *Big Data)* (i.e. data not collected in experimental conditions) [24] for surveillance and/or prediction of dengue outbreaks.

### Exclusion criteria

- Studies without original data, such as reviews, editorials, guidelines and perspectives articles
- Randomized control trials, case series, and case reports
- Descriptive epidemiological studies without any modeling
- Studies on other arbovirus types (e.g. chikungunya, Zika virus disease)
- Studies exclusively on mosquitoes (without any human data) and *in vitro* studies
- Studies only on incidence using geographic information systems

### Search methodology

#### Information sources and search strategy

The literature search was carried out in MEDLINE (PubMed), Scopus and Web of Science between January 1, 2000 and August 31, 2020 to identify potentially eligible studies. MeSH terms and keywords were used to perform the queries. First, the MeSH term “Dengue” was combined with several other MeSH terms (e.g. Data mining, Big Data, Forecasting, Social media), using the Boolean operator AND. Then, a more specific combination of keywords was used for all databases: i) Dengue AND [Monitoring OR Surveillance] AND [Big Data OR Data mining OR Instagram OR Facebook OR Twitter OR Tweets OR Google OR Baidu OR Google Trends OR Social media OR Social network OR Internet], ii) Dengue AND [Prediction OR Forecasting OR Modeling OR Modelling] AND [Big Data OR Data mining OR Instagram OR Facebook OR Twitter OR Tweets OR Google OR Baidu OR Google Trends OR Social media OR Social network OR Internet], iii) Dengue AND [Big Data OR Data mining OR Instagram OR Facebook OR Twitter OR Tweets OR Google OR Baidu OR Google Trends OR Social media OR social network OR Internet].

Relevant articles were also searched in the grey literature, including French-language studies on HAL (Hyper Articles en Lignes) [25], which is an open archive where authors can deposit scholarly documents from all academic fields, *theses*.*fr* [26], which is the French open database for all ongoing and defended PhD theses in France, and the WHO Dengue Bulletin.

Finally, the references of the retained studies and of major dengue epidemiological review articles were screened to identify studies overlooked by the previous search strategies.

#### Selection process

Two independent authors (ES and CJ) screened the title and abstract to select relevant studies for the review. They read the full text of all studies that seemed to meet the eligibility criteria, or if the abstract was not explicit enough to make a decision. In case of disagreement, a third reviewer helped to reach a consensus (AC).

#### Quality assessment, data collection, extraction, and analysis

Two reviewers (ES and CJ) extracted data from the selected articles, including first and last authors, year of publication, study period, objectives, study population, methodology, model performance and evaluation, study site (S1 Text).

As reporting guidelines for machine learning models and real-world data studies are not available, each reviewer independently performed a quality assessment using quality assessment criteria described in previous review articles on these topics [27–29] (S1 Table). A narrative synthesis of all eligible studies was prepared using the following framework: i) data sources and outcomes, ii) statistical and machine learning methods, iii) evaluation metrics, and iv) study results.

All descriptive analyses from the extracted articles were performed using R version 3.6.3 [30].

## Results

**Fig 1:**
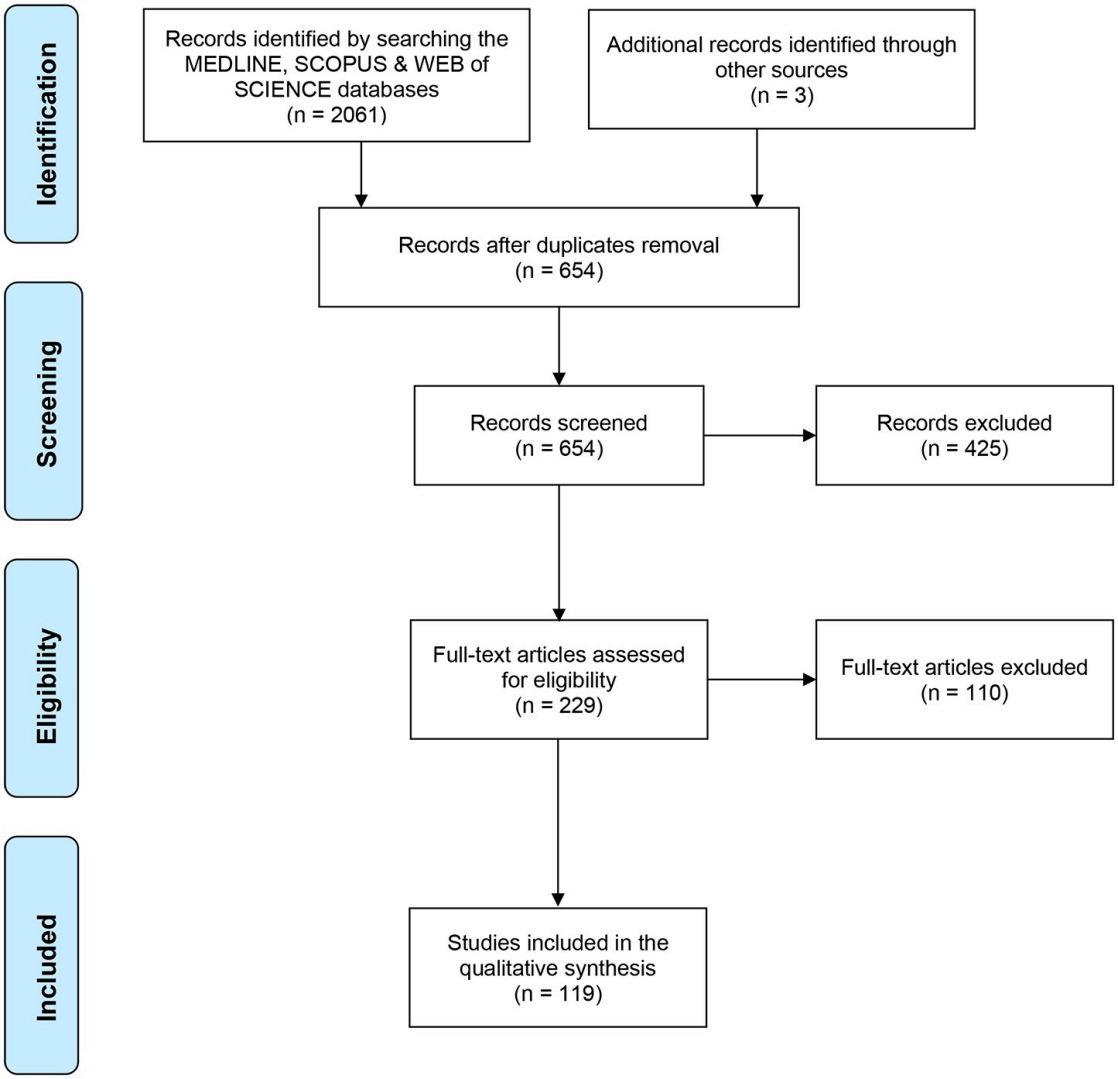
PRISMA Flow Diagram describing the screening process for the systematic review.

**Fig 2:**
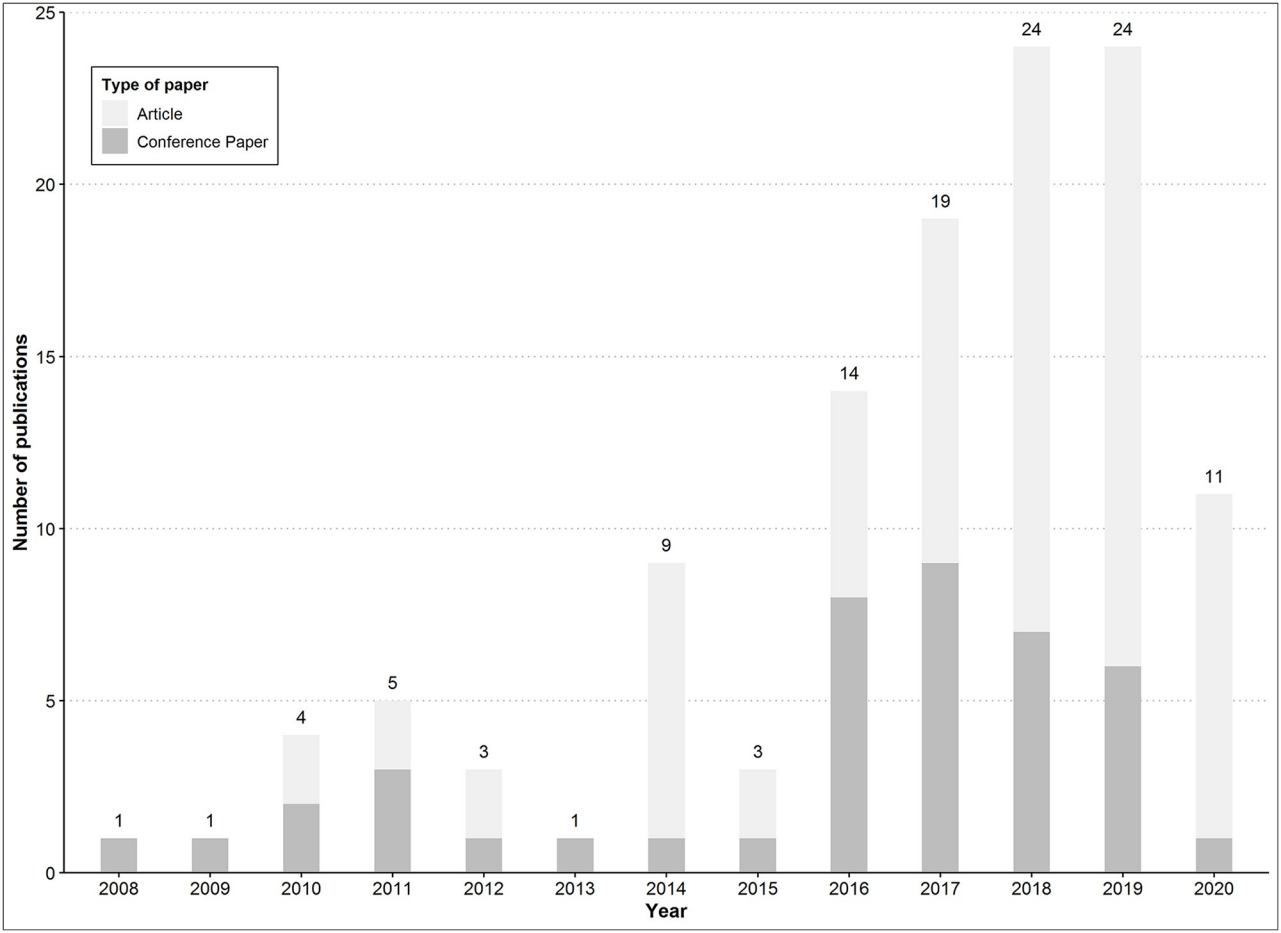
Number of publications on dengue prediction and/or surveillance published between January 1, 2000 and August 31, 2020.

Among the 2064 studies identified, 119 articles were included in this systematic review (Fig 1) [31–148]. Although the search time window was from January 1, 2000, the first included studies were published in 2008, and 67% of the eligible articles were published between 2016 and 2020 (Fig 2). The study populations were predominantly from South-East Asia (37%) and South America (22%). Among the 119 papers included, 77 (65%) were articles, and 42 (35%) were conference papers. On the basis of the Web of Science “Research Area” and the Scopus “Subject Area” classification, the topic of the selected articles were aggregated into eight categories and three main themes: i) Information Technology & Science (52% of all articles), ii) Medicine (24%), and iii) Health Informatics, Public Health & Biology (24%) (Table 1). Conference papers were mainly classified in the “Information Technology & Science” category (39/42; 93%), whereas articles were more evenly distributed in the “Medicine” (28/77; 36%), “Health Informatics, Public Health & Biology” (26/77; 34%) and “Information Technology & Science” (23/77; 30%) themes (S2 Table). The complete list of all selected studies and their characteristics are in S3 Table.

**Table 1:** Type, study population and themes of the selected studies.

	n	%
**Study type**	**119**	
Article	77	65
Conference paper	42	35
**Geographic region** *		
Americas		
Caribbean	3	2
North America	3	2
South America	28	22
Asia		
East Asia	16	13
South-East Asia	47	37
South Asia	27	21
Australia	1	1
Worldwide	2	2
**Study main theme**		
Information Technology & Science	**62**	**52**
Computer Science	42	35
Engineering	10	8
Science & Technology—Other Topics	10	8
Medicine	**28**	**24**
Infectious Diseases & Tropical Medicine	20	17
Medicine—Other Topics	8	7
Health Informatics, Public Health & Biology	**29**	**24**
Biology	7	6
Medical Informatics	16	13
Public Health	6	5

*Some studies were carried out in more than one geographic regions

### Data sources

All included studies, except one [68], used only retrospective data. Most articles had multiple and heterogeneous data sources. The most conventional data sources were: government agencies (n = 72, 46%) and medical institutions (e.g. hospitals/laboratories) (n = 30, 19%). The data retrieved from these sources included epidemiological data, climate and environmental data from meteorological departments, and clinical and biological data. Some studies also used open access data from the WHO or from databases of published studies (S3 Table).

Among the included studies, 47/119 (39%) used at least one novel data stream, such as Internet search engines and social networks [14]. Most of these studies (n = 41, 87%) were published after 2015. Google was the most frequently used Internet search engine (n = 19 studies) and Twitter the most frequently used social network (n = 18). Many studies based on novel data streams were research articles (n = 33, 70%), but the main theme, regardless of the study type (Conference paper or Article) varied depending on the data. Specifically, studies based on Google data were classified homogeneously into the three main themes. Conversely, studies that exploited social networks as data source were evenly distributed between Conference papers (n = 9) and Articles (n = 10), but only few of them were classified into the Medicine theme (Table 2).

**Table 2:** Data sources for dengue monitoring and prediction depending on the main theme.

Number of studies n(%)^a^			Study main theme n (%)	
		IT	Med	PH
	**119**	**62**	**28**	**29**
**Traditional data sources**				
Epidemiological and demographic data	86 (72)	42 (68)	24 (86)	20 (69)
Clinical and biological data	33 (27)	20 (32)	3 (11)	10 (34)
Genomic sequence data	2 (1)	1 (2)	0 (0)	1 (3)
Climate, environmental and geographic data	45 (37)	26 (42)	12 (43)	7 (24)
Vector data	4 (3)	1 (2)	3 (11)	0 (0)
**Novel data streams**				
**Internet search engine data**	25 (21)	8 (13)	11 (39)	6 (21)
Baidu	6 (5)	2 (3)	4 (14)	0 (0)
Google	19 (15)	6 (9)	7 (25)	6 (20)
**Social media data**	21 (17)	14 (22)	4 (14)	3 (10)
Twitter	18 (14)	12 (19)	4 (14)	2 (6)
Other	3 (2)	2 (3)	0 (0)	1 (3)
**Other data sources**	10	2 (3)	3 (11)	5 (17)
Cellphone	2	2 (3)	0 (0)	0 (0)
HealthMap	2	0 (0)	1 (3)	1 (3)
LeXisNexis	2	0 (0)	1 (3)	1 (3)
Political stability	1	0 (0)	0	1 (3)
Wikipedia	1	0 (0)	1 (3)	0 (0)

^a^ As most studies used several data sources, some articles are present several times.

IT: Information Technology & Science; Med: Medicine; PH: Health Informatics, Public Health & Biology

Most studies used structured data, but 41 (34%) studies had an unstructured data source, such as Internet search-based queries or Twitter (Table 2). Among the 41 studies that used unstructured data, 28 (68%) did not develop their own pre-processing methods for these data sources, but simply used keywords related to their research. However, when studies used Natural-Language Processing (NLP)-based methods, they had a full pre-processing framework based on the NLP state-of-the-art recommendations.

Overall, studies that used non-conventional data relied less frequently on clinical data. Conversely, studies that used human data relied mostly on traditional sources, such as weather and environmental data. Moreover, genomic and vector data were vastly underused in combination with other sources, because only five studies using at least one of these sources were included in this systematic review. Data sources are detailed in Table 2.

### Statistical methods

The main aim of the included studies was to predict a dengue-related outcome (n = 65, 55%), to assess the validity of data sources for dengue surveillance (n = 29, 24%), or both (n = 25, 21%). The most frequently chosen outcomes (for prediction and monitoring) were dengue incidence rate (n = 58, 49%), dengue diagnosis based on symptoms (n = 20, 17%), and dengue outbreaks (n = 18, 15%) (S4 Table).

Only one study [48] used NLP-based methods for dengue prediction or surveillance, but as a pre-treatment step to extract and format data for modelling.

The model choice was related to the study objectives (prediction/forecasting or validity of a data source for dengue monitoring). Overall, most studies compared the performances of different models and statistical methods. The most frequently used models, regardless of the study aim(s), were regression-based models (25%), followed by decision-tree models (18%), and artificial neural networks (15%). Most studies on dengue monitoring used correlation analyses to identify relevant variables and/or data sources. Correlation methods (Pearson correlation or Spearman correlation) were especially useful to assess the validity of novel data streams, such as Twitter and Internet search engines. Most studies that included machine-learning algorithms used supervised learning methods (69%). The models’ characteristics are detailed in Table 3.

**Table 3:** Statistical methods and models used in the selected studies depending on the study aim*.

Statistical methods	Prediction n (%)	Surveillance ^a^ n (%)	Prediction and surveillance n (%)	Totaln (%)
**Methods for statistical analysis**	**153**	**59**	**68**	**280**
Machine learning methods	126 (82)	27 (46)	51 (75)	**204 (73)**
Supervised learning	121 (79)	21 (36)	50 (74)	**192 (69)**
Unsupervised learning	5 (3)	6 (10)	1 (1)	**12 (4)**
Other model types (including time series models)	25 (16)	9 (15)	4 (6)	**38 (14)**
Correlation	2 (1)	23 (39)	13 (19)	**38 (14)**
**Models for analyses**	**151**	**36**	**55**	**242**
Artificial neural networks	31 (21)	2 (6)	3 (5)	**36 (15)**
Association rules	3 (2)	1 (3)	0 (0)	**4 (2)**
Bayesian models	12 (8)	5 (14)	3 (5)	**20 (8)**
Clustering	5 (3)	5 (14)	1 (2)	**11 (5)**
Decision tree	35 (23)	2 (6)	6 (11)	**43 (18)**
Regression model	20 (13)	9 (25)	31 (56)	**60 (25)**
Support-vector machine	17 (11)	3 (8)	7 (13)	**27 (11)**
Time series	12 (8)	1 (3)	3 (5)	**16 (7)**
Other ^b^	16 (11)	8 (22)	1 (2)	**25 (10)**

*As most studies used several models and/or statistical methods, some are listed several times.

^a^ Studies evaluating a data source (traditional or novel data streams) for dengue monitoring

^b^ Some models classified as “Other” are also included in the “Supervised learning” category

To evaluate and assess the performance of the chosen statistical methods and/or models, 71 studies (60%) used a machine learning approach and partitioned their data into a training set and a test set. Like for the models, the choice of evaluation metrics was closely related to the study aim(s). All articles used at least one metric, and most of them more than one. Overall, the most common metrics were based on a Confusion Matrix (53%), with Accuracy as the most used metric, followed by Recall or Sensitivity. Correlation-based metrics were used in 37% of studies, especially correlation coefficients (Pearson or Spearman, depending on the data source). The aim of most studies that used correlation metrics was to assess a data source for dengue monitoring (n = 37, 84% of the 44 studies with correlation metric). Error-based metrics were also commonly used (n = 35, 29% of all studies). Few studies used other metrics (n = 22, 18% of all studies) and only 9 studies (8%) did not used at least one metrics falling into the above categories. (Table 4).

**Table 4:** Evaluation metrics used in the selected articles depending on their aim(s)*.

Evaluation metrics	Prediction n (%)	Surveillance ^a^ n(%)	Prediction and surveillance n(%)	Total n(%)
**Correlation metrics**	**8**	**22**	**18**	**48**
Correlation coefficient	3 (38)	16 (73)	9 (50)	**28 (58)**
R-squared	4 (50)	5 (23)	9 (50)	**18 (38)**
Other correlation metric	1 (12)	1 (5)	0 (0)	**2 (4)**
**Error-based metrics**	**34**	**2**	**21**	**57**
Root mean square error	14 (41)	0 (0)	9 (43)	**23 (40)**
Mean absolute error	7 (21)	0 (0)	4 (19)	**11 (19)**
Mean absolute percentage error	4 (12)	0 (0)	3 (14)	**7 (12)**
Mean squared error	3 (9)	0 (0)	3 (14)	**6 (11)**
Other	6 (18)	2 (100)	2 (10)	**10 (18)**
**Confusion matrix-based metrics**	**147**	**13**	**17**	**177**
Accuracy	38 (26)	6 (46)	7 (41)	**51 (29)**
Recall/Sensitivity	32 (22)	2 (15)	3 (18)	**37 (21)**
Specificity	20 (14)	0 (0)	3 (18)	**23 (13)**
Precision/Positive predictive value	17 (12)	1 (8)	1 (6)	**19 (11)**
F-score	12 (8)	2 (15)	0 (0)	**14 (8)**
AUC and/or ROC curve^b^	16 (11)	1 (8)	3 (18)	**20 (11)**
Kappa statistic	5 (3)	0 (0)	0 (0)	**5 (3)**
Other	7 (5)	1 (8)	0 (0)	**8 (5)**
**Other evaluation metrics**	**10**	**6**	**11**	**27**
**Number of articles using the evaluation metric**	**65**	**29**	**25**	**119**
Correlation metrics	7 (11)	19 (66)	18 (72)	**44 (37)**
Error-based metrics	19 (29)	1 (3)	15 (60)	**35 (29)**
Confusion matrix-based metrics	47 (72)	9 (31)	8 (32)	**64 (54)**
Other evaluation metrics	8 (12)	6 (21)	8 (32)	**22 (18)**

*As studies used several metrics, some articles are listed more than once.

^a^ Studies evaluating a data source (traditional data or novel data streams) for dengue monitoring

^b^ AUC: Area Under the ROC Curve. ROC: Receiver Operating Characteristic

### Study results

Among the 54 studies on surveillance, 37 (68%) assessed novel data streams, such as Internet search engines and social media, particularly Google (n = 16, 30%) and Twitter (n = 16, 30%). The most common traditional data source evaluated was climate, environmental and geographic data (n = 13/54; 24%) (S5 Table). All studies found a statistically significant association between the data source and the dengue-related outcome.

The aim of the studies on prediction (n = 90) could be categorized in two main groups: i) comparing different models to predict a dengue-related outcome, and ii) finding the significant predictors among several covariates in a model. Twenty-two studies (24%) included tried to respond to both aims.

The most significant predictors were rainfall (22 models, 43% of 51 studies), temperature (21 models, 41% of 51 studies), and humidity (13 models, 25% of 51 studies). These predictors were also the most frequent in studies to predict dengue incidence rates or dengue outbreaks. Conversely, in studies on dengue diagnosis prediction, the most frequent predictors were fever (4 models, 66% of 6 studies), arthralgia/myalgia (3 models, 50% of 6 studies), platelet count (2 models, 33% of 6 studies), and white blood cell count (2 models, 33% of 6 studies) (Table 5).

**Table 5:** Most significant predictors for the three most frequently studied outcomes.

Number of studies n (%)	Dengue incidence rates n = 27	Dengue outbreaks n = 9	Dengue diagnosis n = 6
**Significant predictors** *			
Rainfall	14 (52)	7 (78)	0 (0)
Temperature	14 (52)	6 (67)	0 (0)
Humidity	9 (33)	1 (11)	0 (0)
Mosquito-related predictor	0 (0)	2 (22)	0 (0)
Google search index	4 (15)	0 (0)	0 (0)
Baidu search index	3 (11)	0 (0)	0 (0)
Tweets	3 (11)	0 (0)	0 (0)
Fever	0 (0)	0 (0)	4 (66)
Arthralgia/myalgia	0 (0)	0 (0)	3 (50)
Platelet count	0 (0)	0 (0)	2 (33)
White blood cell count	0 (0)	0 (0)	2 (33)
Other	13 (48)	6 (67)	5 (83)

*Most studies found several significant predictors

Overall, in studies comparing different models, neural networks and decision trees gave the best performances and were the best models in 13 studies (52% of 54 studies), followed by support vector machine (9/54 studies, 17%). In studies to predict dengue incidence rates, regression-based models showed the highest performance (5/24 studies, 21%) (Table 6). The full list of models and predictors, depending on the outcome, is provided in S5 Table.

**Table 6:** Model with the best performance for the three most frequently studied outcomes.

Number of studies	Dengue incidence rates n = 24	Dengue outbreaks n = 9	Dengue diagnosis n = 14
**Best model**			
Artificial neural network	4 (17)	1 (11)	4 (29)
Decision tree	4 (17)	2 (22)	4 (29)
Support vector machine	4 (17)	1 (11)	4 (29)
Regression model	5 (21)	1 (11)	0 (0)
Time series	3 (12)	2 (22)	0 (0)
Bayesian models	2 (8)	0 (0)	0 (0)
Association rules	1 (4)	1 (11)	0 (0)
Clustering	0 (0)	0 (0)	1 (7)
Other	1 (4)	1 (11)	1 (7)

## Discussion

This systematic review showed that in the last 20 years, data-driven methods for dengue monitoring and prediction have become very popular, particularly in Asia where 72% of the included studies were performed. Very few studies were carried out outside Asia or the Americas, which is to be expected, because these are the two biggest dengue-endemic regions and 70% of the actual dengue burden is in Asia [149–151]. Studies in African countries were noticeably absent, although this continent also is a dengue-endemic region.

The most frequent data sources were conventional data traditionally used in dengue-related studies, such as case counts, climate, environmental, and clinical data. However, this review also highlighted the growing interest by the scientific community for novel Big Data streams for dengue surveillance and prediction [14,33,39–41,43,49,51–53,56,60,65,66,69–71,75–77,79–81,84,85,91,92,98,100,102,105,110–112,114,115,126,127,130,135–138]. Indeed, social media and Internet search engines have become widely accessible worldwide, and therefore they represented the most popular novel data streams in the included studies. The easy access to these sources facilitates the assessment of their influence on infectious disease surveillance and prediction [152–154]. This is particularly true for neglected tropical diseases, such as dengue, Zika virus disease and chikungunya, because of their reoccurrence and the massive increase of their incidence in recent years [155,156]. Moreover, harnessing these novel data streams can improve traditional dengue surveillance systems, because they allow the early detection of an outbreak, and thus can decrease delays between the actual dengue outbreak onset and the official case notifications [157,158]. In the case of dengue control, early response is especially important because it can influence the outbreak severity.

Our analysis also identified the underutilization of some data sources. Genomic data and vector-based data were exploited only in 6 of the 119 included studies [35,42,50,57,75,131], despite the importance of vector surveillance in dengue. Moreover, studies using genomic data were based only on human genome data, although scientists could easily access viral genome sequencing data, for instance via the European Virus Archive—GLOBAL (EVAg) [159]. EVAg aim is to offer access to viruses and to virus sequencing data (including dengue) to scientists, government agencies and academic institutions. None of the included studies made use of data provided by this archive. The lack of vector data is surprising because this type of information is crucial in dengue monitoring studies[160,161]. However, we could not evaluate publication bias, especially in the case of underused data sources. As all included studies on the pertinence of a data source found a significant association between the source and a dengue-related outcome, we cannot exclude that some data sources were not underused, but rather not relevant for dengue management. However, the nature of the underused data sources could suggest that there is a dichotomy between data sources and the objectives of dengue studies: the studies focus either on techniques for vector monitoring/prediction or on techniques for human surveillance/prediction, but rarely on both. This dichotomy was also observed within human surveillance and prediction studies. Specifically, health scientists seemed to rely mainly on traditional data, whereas information technology researchers focused more on non-traditional data (especially social networks). Thus, studies using hospital data for dengue prediction rarely leveraged other data sources, such as climate data. Conversely, studies based on non-traditional data sources rarely used human data, besides the official number of dengue case counts. This might be explained by the fact that clinical data are often hard to access for researchers, particularly outside the medical community, for legal and ethical reasons. Furthermore, a substantial number of the selected papers were conference papers from Information Technology & Sciences Conferences rather than Medicine Conferences. This might reflect the lack of interactions between research teams focused on prediction and/or informatics and physicians and/or government agencies focused on infectious disease monitoring and management. Yet, this research field would greatly benefit from combining their complementary approaches/expertise. Nevertheless, the most commonly studied outcomes in these articles based on real-world data were dengue incidence rate, dengue outbreaks and dengue diagnosis because they need to assess the reliability of novel data streams compared with traditional data sources. As most studies could demonstrate that these sources and methods can complete traditional surveillance and prediction methods, stakeholders should be more aware of these alternative methodologies and novel data streams, and reach out to these highly specialized teams to optimize outbreak dynamic tracking and to improve data completeness and prediction model accuracy.

Most of the included studies relied on machine learning methods, particularly supervised learning models, to assess traditional and also novel data streams. These models were useful also for the analysis of traditional data sources, and allowed scientists to harness non-structured data with NLP methods [40,43,48,49,51–53,56,60,65,66,69–71,73,76,77,79–81,84,85,92,98,100,102,105,110–112,114,115,126,127,130,134–139]. Unsupervised learning models were not the method of choice in most studies, possibly because these studies wanted to identify relevant data sources and/or indicators for dengue monitoring and prediction. Indeed, unsupervised learning tends to be used to identify clusters with similar characteristics [162,163]. Studies that used these methods wanted to predict dengue diagnosis based on the patient clinical profiles or to assess the validity of novel data sources, such as Twitter. Moreover, this approach for dengue research is fairly recent: with the exception of one conference paper from 2011, all studies using unsupervised learning models were published after 2016. Similarly, most studies relying on NLP methods were published rather recently, especially after 2017 (35 of the 42 studies with NLP methods). These two observations suggest that unsupervised learning and NLP might become more prominent in dengue research. It is important to note that despite the use of real-world data, these statistical methods were employed to analyze only retrospective data (but for one study), making their pertinence in real conditions difficult to assess.

Evaluation metrics are crucial in real-world data studies because they help to determine whether the collected data are fit for the purpose (here, dengue surveillance and prediction) and to assess data quality and bias [164]. Although most of the included prediction studies used at least one of the gold standard metrics for information retrieval, such as precision (or positive predictive value) and recall (or sensitivity) [165], several articles employed only error-based metrics, such as root mean square error and mean absolute error. The choice of evaluation metrics is obviously related to the study objective, but even studies where information retrieval metrics could be calculated did not necessarily use them. Again, these methodological choices might be explained by the discrepancy between health scientists who prefer “traditional” modeling evaluation metrics and information technology scientists who focus on information retrieval metrics.

This study also highlighted that despite the variety of approaches to predict dengue outcomes, some factors are constantly relevant, regardless of the study period or country, such as weather-based predictors, artificial neural networks, and decision tree models. However, a consensus on universal models and data sources has not been reached and will probably be difficult to attain due to the complex nature of dengue transmission.

This review has two main weaknesses despite the systematic approach. First, we only searched for published articles and did not look for preprints. Second, besides the experts involved in this review, we could not obtain the opinion of other international experts due to the infectious disease context of 2020 (COVID-19 and dengue outbreaks in many regions). Therefore, we may have missed relevant studies for the review. Finally, the definition of real-world data can vary according to the stakeholders’ view. We had to choose one single definition for the reviewing process, but other definitions do exist. Therefore, we cannot rule out a selection bias in our study.

Overall, this review showed that combining novel real-world and *Big Data* sources with machine learning methods is a promising approach to improve dengue prediction and outbreak monitoring. These new approaches are especially relevant because they can help government agencies and experts to better prepare for each resurgence and better manage outbreaks. Their aim is not to replace existing systems, but to complement them, especially for reducing delays between outbreaks and reporting. Future studies should focus on better integrating all available data sources and methods to improve the stakeholders’ response and to better understand dengue outbreaks.

## Supporting information

S1 Checklist PRISMA Checklist. (DOCX)

S2 Checklist PRISMA for Abstracts Checklist. (DOCX)

S1 Text Data extraction sheet. (DOCX)

S2 Text PROSPERO Protocol. (PDF)

S1 Table Quality assessment criteria. (DOCX)

S2 Table Themes associated with the included studies. (DOCX)

S3 Table Characteristics of studies included in the systematic review. (DOCX)

S4 Table Studied outcomes in dengue fever surveillance and prediction. (DOCX)

S5 Table Detailed study outcomes and results. (XLSX)
