# Supplementary material for: Data-driven methods for dengue prediction and surveillance using real-world and Big Data: A systematic review
Source: PLoS Negl Trop Dis. 2022 Jan 7;16(1):e0010056. doi: 10.1371/journal.pntd.0010056 (PMC8740963; doi:10.1371/journal.pntd.0010056)
Supplement: S1 Text — (DOCX) [file pntd.0010056.s003.docx]

**S1 Text. Data extraction sheet**

| **Study characteristics** |  |
| --- | --- |
| Reviewer’s initials |  |
| Study ID |  |
| PMID |  |
| DOI |  |
| Study title |  |
| Authors (all of them) |  |
| First author | Last name and First Initial |
| Last author | Last name and First Initial |
| Journal |  |
| Year |  |
| Type of publication | Article or Conference Paper |
|  |  |
| **Study location** |  |
| Continent(s) where the study was performed (select all that apply) | Asia  Americas  Australia  Europe  Australia  Worldwide |
|  |  |
| Region(s) where the study was performed (select all that apply) | Australia  Caribbean  East Asia  Europe  North America  South America  South Asia  South-East Asia  Worldwide |
|  |  |
| Country(ies) where the study was performed (specify the country) |  |
|  |  |
| **Data sources** |  |
| Year(s) of data collection |  |
|  |  |
| Used data sources (select all that apply) | Epidemiological and demographic data  Clinical and biological data  Genomic sequencing data  Climate, environmental and geographic data  Vector data  Internet search engine data (specify)  Social media data (specify)  Other source (specify) |
| Data origin (select all that apply) | Government agency  Hospital  Internet search engine  Public dataset  Social network  World Health Organization  Other source (specify) |
|  |  |
| **Methods** |  |
| Did the study evaluate a data source for monitoring? | Yes  No  Not Specified  Unsure |
|  |  |
| Did the study predict a dengue-related outcome? | Yes  No  Not Specified  Unsure |
|  |  |
| What were the dengue-related outcomes? (specify) |  |
|  |  |
| What algorithms and/or statistical models were used in the study? |  |
|  |  |
| Did the study use a machine learning algorithm? | Yes  No  Not Specified  Unsure |
|  |  |
| If the study used a machine learning algorithm, did it use supervised learning? | Yes (specify)  No  Not Specified  Unsure |
|  |  |
| If the study used a machine learning algorithm, did it use unsupervised learning? | Yes (specify)  No  Not Specified  Unsure |
|  |  |
| If the study did not use a machine learning algorithm, did it use another type of model? | Yes (specify)  No  Not Specified  Unsure |
|  |  |
| Did the study use a natural language processing method? | Yes (specify)  No  Not Specified  Unsure |
| **Evaluation** |  |
| Did the study use separate sets to train and test the algorithms? | Yes  No  Not Specified  Unsure  Not applicable |
|  |  |
| What were the evaluation metrics? (specify) |  |
