# Supplementary material for: Data-driven methods for dengue prediction and surveillance using real-world and Big Data: A systematic review
Source: PLoS Negl Trop Dis. 2022 Jan 7;16(1):e0010056. doi: 10.1371/journal.pntd.0010056 (PMC8740963; doi:10.1371/journal.pntd.0010056)
Supplement: S2 Text — (PDF) [file pntd.0010056.s004.pdf]

S2 Text. PROSPERO Protocol

Amendments to information provided at registration or in the protocol.

Searches:

Inclusion criteria: Publication date changed from 2019 to August 31 2020

Intervention(s), exposure(s)

We expanded the scope of data and included studies with real-world data in general (*Big Data* is a form of real-world data).

Risk of bias (quality) assessment

We expanded our method:

Each reviewer will independently assess the statistical models used in the study. In case of disagreement a third reviewer will help choose the right classification. Regarding prediction, when possible, risk of bias will be assessed using the Prediction model Risk Of Bias AssessmenT (PROBAST). If not (especially for machine learning methods) each reviewer will assess at least each of the following elements: predictor, outcome evaluation metrics.

PROSPERO
International prospective register of systematic reviews

Harnessing Big Data and machine learning methods for dengue surveillance and prediction: a systematic review

Emmanuelle Sylvestre, Marc Cuggia, André Cabié, Clarisse Joachim

To enable PROSPERO to focus on COVID-19 registrations during the 2020 pandemic, this registration record was automatically published exactly as submitted. The PROSPERO team has not checked eligibility.

Citation

Emmanuelle Sylvestre, Marc Cuggia, André Cabié, Clarisse Joachim. Harnessing Big Data and machine learning methods for dengue surveillance and prediction: a systematic review. PROSPERO 2020 CRD42020172472 Available from: https://www.crd.york.ac.uk/prospero/display_record.php?ID=CRD42020172472

Review question

1. What are the different Big data and machine learning methods used for dengue prediction?
2. What are the data sources used?
3. Are these methods reproducible?

Searches

We will search articles in PubMed, Web of Science, Scopus and LILACS.
We will also search studies from grey literature, including the World Health Organization (WHO) Dengue Bulletin.
There will be no language restriction.
We will look for articles published between 2000 and 2019.

Types of study to be included

We will include all epidemiological studies that use Big Data methods to predict: dengue outbreaks, dengue outcomes, dengue severity.
Studies with no original data (reviews, editorials, guidelines, perspective pieces), randomized controlled trials, case series and case reports will not be included.
Descriptive epidemiological studies without any prediction model will not be included.
Studies focusing on other types of arboviruses will not be included.
We will not include studies focusing exclusively on mosquitoes or in vitro studies.

Condition or domain being studied

We will focus on Dengue virus (DENV) which is one of the most important vector-borne diseases in the world.
The majority of DENV infections are asymptomatic or are characterized by intense flu-like symptoms lasting up to 10 days afterward but they can evolve into the severe forms of dengue hemorrhagic fever/dengue shock syndrome (DHF/DSS) which can lead to death. However, mortality due to dengue can be greatly reduced by early diagnosis, which will influence appropriate clinical management.

Most dengue-endemic regions (South-East Asia, the Americas and the Pacific for the most seriously affected) rely on traditional surveillance, based on hospital syndromic monitoring and laboratory confirmation of a subset of cases reported to a central health agency. While this method is generally very accurate; it can be very slow and expensive due to the time needed to aggregate data, with substantial delays between an event and notifications
On the other hand, numerous studies have successfully used mobile, digital and Internet based systems to crowd-source data from the community. These new sources of data have been already used in pilot studies to improve monitoring and clinical management and predict dengue outbreaks.

Participants/population

We will include all people with dengue, regardless of age, gender or severity of the disease.

Intervention(s), exposure(s)

We are interested in studies using Big Data methods. According to the MeSH definition of Big Data, this means all methods applied on “extremely large amounts of data which require rapid and often complex computational analyses to reveal patterns, trends, and associations, relating to various facets of human and non-human entities”.
Regarding machine-learning methods, it can be defined as any computer-derived mathematical algorithm using learning to classify data. It includes:
- Supervised machine learning
- Unsupervised machine learning
- Deep learning

Comparator(s)/control

Not applicable

Context

The diagnostic of dengue in the included papers should be established using any of the standard WHO definition and classifications (1997 or 2009 WHO classification).
Research in low and middle-income countries will also be included.

Main outcome(s)

- Number and type of Big Data methods and/or machine learning models used to predict or forecast a dengue outbreak and their performance (Recall, Precision, F-measure)
- Number and type of Big Data methods and/or machine learning models used to predict or forecast a severe dengue outbreak and their performance (Recall, Precision, F-measure)

Measures of effect

Not applicable

Additional outcome(s)

None

Measures of effect

Not applicable

Data extraction (selection and coding)

Two authors from the review team will independently extract outcome data from each study using a Microsoft Excel collection form. In case of disagreement, a third reviewer will help to reach a consensus.

The data collection and extraction will proceed as follows.
1. Study screening based on title/abstract for each source
2. Removing duplicates
3. Eligibility based on full text review
4. Data extraction with collection form

The collection form will be based on relevant items from the Critical Appraisal and Data Extraction for Systematic Reviews of Prediction Modelling Studies (CHARMS) checklist

Based on the inclusion criteria and the CHARMS cheklist, the data extracted will include:
1. Study characteristics (title, authors, year of publication, journal...)
2. Participants characteristics (age, gender...)
3. Data sources used for the models
4. Outcomes predicted
5. Machine learning models employed and role in the study
6. Models performance and evaluation

We will use Zotero for managing the search and writing the review.
The extracted data will be stored in Microsoft Excel individually.

Risk of bias (quality) assessment

Each reviewer will independently assess risk of bias using the Prediction model Risk Of Bias ASsessment Tool (PROBAST).

Strategy for data synthesis

We will summarise the results using descriptive statistics and a narrative synthesis.
For the narrative synthesis, we will identify common patterns and compile the results into sub categories:
- Most frequent methods used (and machine learning category)
- Performance and evaluation of each model
- Most frequent data sources used
- Contribution of non-clinical data versus clinical data
- Influence of study participants on the model performance (scientific outcomes?)

No meta-analysis will be conducted for this review.

Analysis of subgroups or subsets

If possible, synthesis will be stratified by:
- country income level (high vs low and middle-income)
- age (adults vs children)
- data sources (traditional data sources vs non traditional data sources)

Contact details for further information

Emmanuelle Sylvestre
emmanuelle.sylvestre@chu-martinique.fr

Organisational affiliation of the review

Martinique University Hospital

Review team members and their organisational affiliations

Dr Emmanuelle Sylvestre. Martinique University Hospital
Professor Marc Cuggia. Rennes University Hospital
Professor André Cabié. Martinique University Hospital
Assistant/Associate Professor Clarisse Joachim. Martinique University Hospital

Collaborators

Professor Raymond Césaire. Martinique University Hospital

Type and method of review

Narrative synthesis, Systematic review

Anticipated or actual start date

09 March 2020

Anticipated completion date

31 August 2020

Funding sources/sponsors

This work is financed through Martinique Univeristy Hospital and Rennes University

Conflicts of interest

Language

English

Country

France

Stage of review

Review Ongoing

Subject index terms status

Subject indexing assigned by CRD

Subject index terms

MeSH headings have not been applied to this record

Date of registration in PROSPERO

28 April 2020

Date of first submission

05 March 2020

Stage of review at time of this submission

The review has not started

Stage	Started	Completed
Preliminary searches	No	No

Stage	Started	Completed
Piloting of the study selection process	No	No
Formal screening of search results against eligibility criteria	No	No
Data extraction	No	No
Risk of bias (quality) assessment	No	No
Data analysis	No	No

The record owner confirms that the information they have supplied for this submission is accurate and complete and they understand that deliberate provision of inaccurate information or omission of data may be construed as scientific misconduct.

The record owner confirms that they will update the status of the review when it is completed and will add publication details in due course.

Versions

28 April 2020

PROSPERO
This information has been provided by the named contact for this review. CRD has accepted this information in good faith and registered the review in PROSPERO. The registrant confirms that the information supplied for this submission is accurate and complete. CRD bears no responsibility or liability for the content of this registration record, any associated files or external websites.
