# Supplementary material for: Data-driven methods for dengue prediction and surveillance using real-world and Big Data: A systematic review
Source: PLoS Negl Trop Dis. 2022 Jan 7;16(1):e0010056. doi: 10.1371/journal.pntd.0010056 (PMC8740963; doi:10.1371/journal.pntd.0010056)
Supplement: S1 Table — (DOCX) [file pntd.0010056.s005.docx]

**S1 Table. Quality assessment criteria**

| **Section** | **Checklist item** | **Page** |
| --- | --- | --- |
| **Title and abstract** | |  |
| Title | For prediction: Identify the study as developing and/or validating a multivariable prediction model, the target population, and the outcome to be predicted. |  |
| Abstract | Provide a summary of objectives, study design, setting, participants, sample size, predictors, outcome, statistical analysis, results, and conclusions. |  |
| **Objective and background** | |  |
| Background | For prediction: Explain the context (including whether diagnostic or prognostic) and rationale for developing or validating the multivariable prediction model, including references to existing models. |  |
| Objectives | 1. Specify the objectives, including (for prediction) whether the study developed and/or or validated the model. |  |
|  | 1. Specify the geographic area of the study. |  |
| **Methods** | |  |
| Source of data | 1. Describe the source of data for dengue infection. |  |
|  | 1. Describe the other sources of data. |  |
|  | 1. Specify the study period. |  |
| Participants  (when applicable) | 1. Specify key elements of the study setting (population, age, gender…). |  |
|  | 1. Describe the eligibility criteria for participants. |  |
| Outcome | Clearly define the study outcome. |  |
| Statistical methods | Specify method or model type, all model-building procedures (including feature selection), and method for internal validation. |  |
|  | Assess whether the statistical methods are appropriate for the study aim. |  |
| Predictors  (when applicable) | Clearly define all predictors used for developing or validating the model, including how and when they were measured. |  |
| Evaluation methods | 1. Specify all measures used to assess statistic and model performance and, if relevant, to compare multiple models. |  |
|  | 1. If applicable, specify all measures to validate the methods and the use of a training and validation set. |  |

**S1 Table.** *(continued)*

| **Section** | **Checklist item** | **Page** |
| --- | --- | --- |
| Results | |  |
| Participants (when applicable) | Describe the number of participants and their characteristics including the number of participants with missing data for predictors and outcome. |  |
| Statistical methods | Describe completely and clearly all results. |  |
|  | Explain how to the use the prediction model (when applicable). |  |
| Discussion | |  |
| Limitations | Discuss the study limitations. |  |
| Interpretation | Give an overall interpretation of the results, considering objectives, limitations, and results from similar studies, and other relevant evidence. |  |
| Implications | Discuss the potential use of the approach and implications for future research. |  |
| Other information | |  |
| Funding | Give the source of funding and the role of the funders in the present study. |  |
| Conflict of interest | Give a conflict of interest statement if necessary. |  |
| Supplementary information | Provide information about the availability of supplementary resources, such as study protocol, Web calculator, and data sets. |  |
