# Supplementary material for: Data-driven methods for dengue prediction and surveillance using real-world and Big Data: A systematic review
Source: PLoS Negl Trop Dis. 2022 Jan 7;16(1):e0010056. doi: 10.1371/journal.pntd.0010056 (PMC8740963; doi:10.1371/journal.pntd.0010056)
Supplement: S2 Table — (DOCX) [file pntd.0010056.s006.docx]

**S2 Table. Themes* associated with the included studies**

| **Study main theme n (%)** | **Article**  **n=77** | **Conference paper**  **n=42** |
| --- | --- | --- |
| **Information Technology & Science** | **23 (30)** | **39 (93)** |
| Computer Science | 12 (16) | 30 (71) |
| Engineering | 5 (6) | 5 (12) |
| Science & Technology – Other Topics | 6 (8) | 4 (10) |
|  |  |  |
| **Medicine** | **28 (36)** | **0 (0)** |
| Infectious Diseases & Tropical Medicine | 20 (26) | 0 (0) |
| Medicine – Other Topics | 8 (10) | 0 (0) |
|  |  |  |
| **Health Informatics, Public Health & Biology** | **26 (34)** | **3 (7)** |
| Biology | 7 (9) | 0 (0) |
| Medical Informatics | 13 (17) | 3 (7) |
| Public Health | 6 (8) | 0 (0) |

*The themes are based on the “Research areas” classification from Web of Science or “Subject areas” from Scopus. Similar themes from both classifications have been aggregated.
