# Supplementary material for: Data-driven methods for dengue prediction and surveillance using real-world and Big Data: A systematic review
Source: PLoS Negl Trop Dis. 2022 Jan 7;16(1):e0010056. doi: 10.1371/journal.pntd.0010056 (PMC8740963; doi:10.1371/journal.pntd.0010056)
Supplement: S3 Table — (DOCX) [file pntd.0010056.s007.docx]

**S3 Table. Characteristics of studies included in the systematic review**

| **Reference** | **Study Type** | **Geographic region** | **Data sources*** | **Research period** | **Origin of data sources**** |
| --- | --- | --- | --- | --- | --- |
| Polwiang et al. (2020)[1] | Article | Thailand | Epidemiological, Climate | 2003-2017 | Government agency |
| Xu et al. (2020)[2] | Article | China | Epidemiological, Climate | 2005-2018 | Government agency |
| Rangarajan et al. (2019)[3] | Article | Singapore, Taiwan,  Thailand, Brazil, Mexico, USA | Epidemiological, Clinical, Google | 2001-2015 | Public dataset |
| Anno et al. (2019)[4] | Article | Taiwan | Epidemiological, Climate | 1998-2015 | Government agency |
| Romero et al. (2019)[5] | Article |  | Epidemiological, Climate, Vector | 1960-2013/  1986-2014 | Public dataset |
| Mello-Román et al. (2019)[6] | Article | Paraguay | Epidemiological, Clinical | 2012-2016 | Government agency |
| Stolerman et al. (2019)[7] | Article | Brazil | Epidemiological | 2002-2017 | Government agency |
| Macedo et al. (2019)[8] | Article | Brazil | Clinical | 2007-2013 | Hospital |
| Husnayain et al. (2019)[9] | Article | Indonesia | Epidemiological, Google | 2012-2016 | Government agency, ISE |
| Souza et al. (2019)[10] | Article | Brazil | Epidemiological, Twitter | 2015 | Government agency, SM |
| Ramadona et al. (2019)[11] | Article | Indonesia | Epidemiological, Twitter | 2016-2018 | Government agency, SM |
| Davi et al. (2019)[12] | Article | Brazil | Clinical, Genomic |  | Hospital |
| Guo et al. (2019)[13] | Article | China | Epidemiological, Climate, Baidu, Weibo | 2011-2016 | Government agency, SM |
| Koh et al. (2018)[14] | Article | Singapore | Epidemiological, Climate | 2016 | Government agency |
| Carvajal et al. (2018)[15] | Article | Philippines | Epidemiological, Climate | 2009-2013 | Government agency |
| Baquero et al. (2018)[16] | Article | Brazil | Epidemiological, Climate | 2000-2016 | Government agency |
| Chen et al. (2018)[17] | Article | Thailand, Taiwan, Singapore | Epidemiological, Climate | 2003-2014 | Government agency |

*Epidemiological: Epidemiological and demographics data; Climate: Climate, environmental and geographical data; Clinical: Clinical and biological data; SM: Social Media data; Vector: Vector data; Genomic: Genomic data; Cellphone: Cellphone data

**ISE: Internet Search Engine; SM: Social Media; WHO: World Health Organization; IoT: Internet of Things

**S3 Table** *(Continued)*

| **Reference** | **Study Type** | **Geographic region** | **Data sources*** | **Research period** | **Origin of data sources**** |
| --- | --- | --- | --- | --- | --- |
| Villanes et al. (2018)[18] | Article | India | LeXisNexis | 2014 | News database |
| Guo et al. (2017)[19] | Article | China | Epidemiological, Climate, Baidu | 2011-2014 | Government agency |
| Chatterjee et al. (2018)[20] | Article |  | Clinical, Genomic |  | Public dataset |
| Guo et al. (2017)[21] | Article | China | Epidemiological, Baidu | 2011-2014 | Government agency, ISE |
| Yang et al. (2017)[22] | Article | Mexico, Taiwan,  Thailand, Brazil, Singapore | Epidemiological, Google | 2001-2015 | Government agency |
| Marques-Toledo et al. (2017)[23] | Article | Brazil | Epidemiological, Twitter, Wikipedia | 2012-2016 | Government agency, SM |
| Premaratne et al. (2017)[24] | Article | Sri Lanka | Clinical | 2016 | Published dataset |
| Jayasundara et al. (2018)[25] | Article | Sri Lanka | Clinical | 2016 | Published dataset |
| Li et al. (2017)[26] | Article | China | Epidemiological, Climate, Baidu | 2011-2014 | Government agency, ISE |
| Kesorn et al. (2015)[27] | Article | Thailand | Epidemiological, Climate, Vector | 2007-2013 | Government agency |
| Dayama et al. (2014)[28] | Article | Singapore | Epidemiological | 2000-2014 |  |
| Sampath et al. (2014)[29] | Article | Singapore | Epidemiological |  | Government agency |
| Gluskin et al. (2014)[30] | Article | Mexico | Epidemiological, Climate, Google | 2003-2011 | Government agency, ISE |
| Flamand et al. (2014)[31] | Article | French Guiana | Epidemiological, Climate | 2006-2011 | Government agency |
| Torres et al. (2014)[32] | Article | Colombia | Epidemiological | 1995-2011/  1997-2011 | Government agency |
| Buczak et al. (2012)[33] | Article | Peru | Epidemiological, Climate, Political stability | 2001-2009 | Government agency |

*Epidemiological: Epidemiological and demographics data; Climate: Climate, environmental and geographical data; Clinical: Clinical and biological data; SM: Social Media data; Vector: Vector data; Genomic: Genomic data; Cellphone: Cellphone data

**ISE: Internet Search Engine; SM: Social Media; WHO: World Health Organization; IoT: Internet of Things

**S3 Table** *(Continued)*

| **Reference** | **Study Type** | **Geographic region** | **Data sources*** | **Research period** | **Origin of data sources**** |
| --- | --- | --- | --- | --- | --- |
| Hoen et al. (2012)[34] | Article |  | Epidemiological, Climate, HealthMap | 2009-2011 | Government agency,  News database |
| Althouse et al. (2011)[35] | Article | Singapore, Thailand | Epidemiological, Google | 2004-2011 | Government agency |
| Chan et al. (2011)[36] | Article | Bolivia, India, Indonesia, Brazil, Singapore | Epidemiological, Google | 2003-2010 | WHO, ISE |
| Faisal et al. (2012)[37] | Article | Malaysia | Clinical, Bioelectrical impedance analysis | 2010 | Hospital |
| Ibrahim et al. (2010)[38] | Article | Malaysia | Clinical, Bioelectrical impedance analysis | 2010 | Hospital |
| Syamsuddin et al. (2020)[39] | Article | Indonesia | Epidemiological, Google | 2012-2017 | Hospital, ISE |
| Romero-Alvarez et al. (2020)[40] | Article | Brazil | Epidemiological, Google | 2011-2016 | ISE, Government agency |
| Liu et al. (2019)[41] | Article | China | Epidemiological, Climate, Baidu | 2011-2015 | Government agency, ISE |
| Musa et al. (2019)[42] | Article | Taiwan | Epidemiological | 2014–2016 |  |
| Messina et al. (2019)[43] | Article | Worldwide | HealthMap | 1960-2015 | HealthMap |
| Titus et al. (2018)[44] | Article | Bangladesh | Epidemiological, Climate | 2000-2009 | Government agency |
| Marques-Toledo et al. (2019)[45] | Article | Brazil | Epidemiological, Climate, Vector, Twitter | 2015 | Government agency, SM |
| Verma et al. (2018)[46] | Article | India | Epidemiological, Google | 2016 | Government agency, ISE |
| Ho et al. (2018)[47] | Article | Philippines | Epidemiological, Google | 2009-2014 | Government agency, ISE |
| Phakhounthong et al. (2018)[48] | Article | Cambodia | Clinical | 2009 | Hospital |
| Strauss et al. (2017)[49] | Article | Venezuela | Epidemiological, Google | 2004-2014 | Government agency |
| Nsoesie et al. (2016)[50] | Article | Brazil | Epidemiological, Twitter | 2012-2014 | Government agency, SM |

*Epidemiological: Epidemiological and demographics data; Climate: Climate, environmental and geographical data; Clinical: Clinical and biological data; SM: Social Media data; Vector: Vector data; Genomic: Genomic data; Cellphone: Cellphone data

**ISE: Internet Search Engine; SM: Social Media; WHO: World Health Organization; IoT: Internet of Things

**S3 Table** *(Continued)*

| **Reference** | **Study Type** | **Geographic region** | **Data sources*** | **Research period** | **Origin of data sources**** |
| --- | --- | --- | --- | --- | --- |
| Liu et al. (2016)[51] | Article | China | Epidemiological, Baidu | 2010-2014 | Government agency, ISE |
| Ximenes et al. (2016)[52] | Article | Brazil | Epidemiological | 2000-2015 | Government agency |
| Mohamad et al. (2014)[53] | Article | Malaysia | Epidemiological, Climate | 2003-2009 | Government agency, Hospital |
| Puengpreeda, et al. (2020)[54] | Article | Thailand | Epidemiological, Climate, Google | 2007-2018 | Government agency, ISE |
| Amin, et al. (2020)[55] | Article | Worldwide | Twitter | 2017-2019 | SM |
| Manogaran, et al. (2018)[56] | Article | India | Epidemiological, Climate | 1979-2016 | Government agency  IoT Weather sensor device |
| Agarwal, et al. (2018)[57] | Article | India | Epidemiological, Climate | 2006-2015 | Government agency |
| Manogaran, et al. (2018)[58] | Article | India | Epidemiological, Climate | 1998-2016 | Government agency,  Public dataset |
| Jahangir, et al. (2018)[59] | Conference Paper | Pakistan | Clinical |  | Hospital |
| Husin, et al. (2018)[60] | Conference Paper | Malaysia | Clinical | 2015 | Hospital, Experts |
| Anggraeni, et al. (2018)[61] | Conference Paper | Indonesia | Epidemiological, Google | 2010-2015 | Hospital, ISE |
| Livelo, et al. (2018)[62] | Conference Paper | Philippines | Epidemiological, Twitter | 2017 | Government agency, SM |
| Wiratmadja, et al. (2018)[63] | Article | Indonesia | Clinical |  | Hospital |
| Arafiyah, et al. (2018)[64] | Conference Paper | Indonesia | Clinical | 2017 | Hospital |
| Abuhamad, et al. (2017)[65] | Article | Malaysia | Epidemiological, Clinical, Climate | 2003-2010 | Government agency |
| Manivannan, et al. (2017)[66] | Conference Paper | Vietnam | Clinical | 2010-2013 | Hospital |

*Epidemiological: Epidemiological and demographics data; Climate: Climate, environmental and geographical data; Clinical: Clinical and biological data; SM: Social Media data; Vector: Vector data; Genomic: Genomic data; Cellphone: Cellphone data

**ISE: Internet Search Engine; SM: Social Media; WHO: World Health Organization; IoT: Internet of Things

**S3 Table** *(Continued)*

| **Reference** | **Study Type** | **Geographic region** | **Data sources*** | **Research period** | **Origin of data sources**** |
| --- | --- | --- | --- | --- | --- |
| Dharmawardana, et al. (2017)[67] | Conference Paper | Sri Lanka | Epidemiological, Climate, Cellphone | 2012-2014 | Government agency,  Cell towers |
| Espina, et al. (2017)[68] | Conference Paper | Philippines | Epidemiological, Twitter | 2016 | Government agency, SM |
| Rahim, et al. (2017)[69] | Article | Malaysia | Epidemiological, Climate | 2010-2015 | Government agency |
| Klein, et al. (2017)[70] | Article | Brazil | Epidemiological, Facebook, Twitter, Instagram, Flickr, YouTube | 2016 | Government agency, SM |
| Kerdprasop, et al. (2016)[71] | Conference Paper | Thailand | Epidemiological, Climate | 2006-2015 | Government agency |
| Anggraeni, et al. (2016)[72] | Conference Paper | Indonesia | Epidemiological, Google | 2010-2015 | Hospital, ISE |
| Mathulamuthu, et al. (2016)[73] | Conference Paper | Malaysia | Epidemiological, Climate | 2009-2013 | Government agency |
| Rahmawati, et al. (2016)[74] | Conference Paper | Taiwan | Epidemiological, Climate | 2014-2015 | Government agency |
| Missier, et al. (2016)[75] | Conference Paper | Brazil | Epidemiological, Twitter | 2015 | Government agency, SM |
| Abeyrathna, et al. (2016)[76] | Conference Paper | Sri Lanka | Epidemiological, Cellphone | 2013 | Government agency,  Cell towers |
| Fathima, et al. (2015)[77] | Article | India | Clinical | 2009-2011 | Hospital |
| Tazkia, et al. (2015)[78] | Conference Paper | Indonesia | Epidemiological, Climate | 2012-2013 | Government agency |
| Wu, et al. (2008)[79] | Conference Paper | Singapore | Epidemiological, Climate | 2001-2006 | Government agency |
| Salam, et al. (2019)[80] | Article | India | Epidemiological, Google | 2004-2017 | Government agency, ISE |
| Saire, et al. (2019)[81] | Conference Paper | Brazil | Twitter | 2009-2017 | SM |

*Epidemiological: Epidemiological and demographics data; Climate: Climate, environmental and geographical data; Clinical: Clinical and biological data; SM: Social Media data; Vector: Vector data; Genomic: Genomic data; Cellphone: Cellphone data

**ISE: Internet Search Engine; SM: Social Media; WHO: World Health Organization; IoT: Internet of Things

**S3 Table** *(Continued)*

| **Reference** | **Study Type** | **Geographic region** | **Data sources*** | **Research period** | **Origin of data sources**** |
| --- | --- | --- | --- | --- | --- |
| Swain, et al. (2017)[82] | Conference Paper | India | Epidemiological, Twitter | 2016 | SM |
| Saravanan, et al. (2017)[83] | Conference Paper | India | Epidemiological, Clinical |  | Hospital |
| Carlos, et al. (2017)[84] | Conference Paper | Brazil | Epidemiological, Twitter | 2015-2016 | Government agency, SM |
| Ye, et al. (2016)[85] | Article | China | Epidemiological, Twitter, Weibo | 2014 | Government agency, SM |
| Li, et al. (2016)[86] | Conference Paper | China | Epidemiological, Climate | 2015 | Government agency |
| Fathima, et al. (2011)[87] | Conference Paper | India | Clinical |  | Hospital |
| Srilekha et al. (2020)[88] | Article | Sri Lanka | Epidemiological, Climate | 18 years |  |
| Ganthimathi et al. (2020)[89] | Article | India | Clinical |  | Hospital |
| Kumar et al. (2020)[90] | Article | India | Clinical | 2017 | Hospital |
| Guiyab et al. (2019)[91] | Article | Philippines | Clinical | 2013-2017 | Hospital |
| Chovatiya et al. (2019)[92] | Conference Paper | India | Epidemiological, Climate |  | Government agency |
| Kerdprasop et al. (2019)[93] | Conference Paper | Thailand | Epidemiological, Climate | 2003-2017 | Government agency |
| Link et al. (2019)[94] | Conference Paper | Peru, Puerto Rico | Epidemiological, Climate | 2002-2009 | Public dataset |
| Arafiyah et al. (2018)[95] | Conference Paper | Indonesia | Clinical | 2017 | Hospital |
| Mishra et al. (2018)[96] | Article | India | Clinical |  | Hospital |

*Epidemiological: Epidemiological and demographics data; Climate: Climate, environmental and geographical data; Clinical: Clinical and biological data; SM: Social Media data; Vector: Vector data; Genomic: Genomic data; Cellphone: Cellphone data

**ISE: Internet Search Engine; SM: Social Media; WHO: World Health Organization; IoT: Internet of Things

**S3 Table** *(Continued)*

| **Reference** | **Study Type** | **Geographic region** | **Data sources** | **Research period** | **Origin of data sources**** |
| --- | --- | --- | --- | --- | --- |
| Wu et al. (2017)[97] | Conference Paper | Taiwan | Epidemiological, Climate, Google | 2006-2016 | Government agency  WHO, ISE |
| Albinati et al. (2017)[98] | Conference Paper | Brazil | Epidemiological, Twitter | 2011-2016 | Government agency, SM |
| Zhu et al. (2017)[99] | Conference Paper | Hong-Kong | Epidemiological, Climate | 2004-2015 | Government agency |
| Zainudin et al. (2016)[100] | Article | Malaysia | Epidemiological | 2010-2015 | Government agency |
| Milinovich et al. (2014)[101] | Article | Australia | Epidemiological, Google | 2004-2013 | Government agency, ISE |
| Ongruk et al. (2014)[102] | Conference Paper | Thailand | Epidemiological, Climate, Vector | 2010-2012 | Government agency |
| Balasundaram et al. (2013)[103] | Conference Paper | India | Clinical |  | Hospital |
| Wu et al. (2009)[104] | Conference Paper | Singapore | Epidemiological, Climate | 2001-2007 | Government agency |
| Zhang et al. (2020)[105] | Article | India | Epidemiological, LeXisNexis | 2003-2016 | WHO, News database |
| Souza et al. (2018)[106] | Conference Paper | Brazil | SM | 2015 | SM |
| Coberly et al. (2014)[107] | Article | Philippines | Epidemiological, Twitter | 2011 | Government agency, SM |
| Gomide et al. (2011)[108] | Conference Paper | Brazil | Epidemiological, Twitter | 2007-2010 | Government agency, SM |
| Fang et al. (2010)[109] | Conference Paper | Taiwan | Epidemiological, Google | 2004-2008 | Government agency |
| Souza et al. (2020)[110] | Conference Paper | Puerto Rico | Epidemiological, Climate, Google | 2004-2008 | Public dataset |
| Yogapriya et al. (2019)[111] | Article | India | Clinical |  | Hospital |

*Epidemiological: Epidemiological and demographics data; Climate: Climate, environmental and geographical data; Clinical: Clinical and biological data; SM: Social Media data; Vector: Vector data; Genomic: Genomic data; Cellphone: Cellphone data

**ISE: Internet Search Engine; SM: Social Media; WHO: World Health Organization; IoT: Internet of Things

**S3 Table** *(Continued)*

| **Reference** | **Study Type** | **Geographic region** | **Data sources*** | **Research period** | **Origin of data sources**** |
| --- | --- | --- | --- | --- | --- |
| Adias et al. (2019)[112] | Conference Paper | Indonesia | Clinical | 2010-2015 | Hospital |
| Jongmuenwai et al. (2019)[113] | Conference Paper | Thailand | Epidemiological, Clinical, Climate | 2007-2016 | Government agency,  Hospital |
| Balasaravanan et al. (2018)[114] | Article | India | Clinical |  | Government agency |
| Acosta et al. (2016)[115] | Article | Cuba | Clinical | 2014 | Hospital |
| Soonthornphisaj et al. (2016)[116] | Conference Paper | Thailand | Clinical |  | Hospital |
| Fathima et al. (2012)[117] | Conference Paper | India | Clinical |  | Hospital |
| Fathima et al. (2011)[118] | Conference Paper | India | Clinical |  | Hospital |
| Long et al. (2010)[119] | Conference Paper | Malaysia | Epidemiological |  | Government agency |

*Epidemiological: Epidemiological and demographics data; Climate: Climate, environmental and geographical data; Clinical: Clinical and biological data; SM: Social Media data; Vector: Vector data; Genomic: Genomic data; Cellphone: Cellphone data

**ISE: Internet Search Engine; SM: Social Media; WHO: World Health Organization; IoT: Internet of Things

# References

1. Polwiang S. The time series seasonal patterns of dengue fever and associated weather variables in Bangkok (2003-2017). BMC Infect Dis. 2020;20: 208. doi:10.1186/s12879-020-4902-6

2. Xu J, Xu K, Li Z, Meng F, Tu T, Xu L, et al. Forecast of Dengue Cases in 20 Chinese Cities Based on the Deep Learning Method. Int J Environ Res Public Health. 2020;17. doi:10.3390/ijerph17020453

3. Rangarajan P, Mody SK, Marathe M. Forecasting dengue and influenza incidences using a sparse representation of Google trends, electronic health records, and time series data. PLoS Comput Biol. 2019;15: e1007518. doi:10.1371/journal.pcbi.1007518

4. Anno S, Hara T, Kai H, Lee M-A, Chang Y, Oyoshi K, et al. Spatiotemporal dengue fever hotspots associated with climatic factors in Taiwan including outbreak predictions based on machine-learning. Geospat Health. 2019;14. doi:10.4081/gh.2019.771

5. Romero D, Olivero J, Real R, Guerrero JC. Applying fuzzy logic to assess the biogeographical risk of dengue in South America. Parasit Vectors. 2019;12: 428. doi:10.1186/s13071-019-3691-5

6. Mello-Román JD, Mello-Román JC, Gómez-Guerrero S, García-Torres M. Predictive Models for the Medical Diagnosis of Dengue: A Case Study in Paraguay. Comput Math Methods Med. 2019;2019: 7307803. doi:10.1155/2019/7307803

7. Stolerman LM, Maia PD, Kutz JN. Forecasting dengue fever in Brazil: An assessment of climate conditions. PLoS One. 2019;14: e0220106. doi:10.1371/journal.pone.0220106

8. Macedo Hair G, Fonseca Nobre F, Brasil P. Characterization of clinical patterns of dengue patients using an unsupervised machine learning approach. BMC Infect Dis. 2019;19: 649. doi:10.1186/s12879-019-4282-y

9. Husnayain A, Fuad A, Lazuardi L. Correlation between Google Trends on dengue fever and national surveillance report in Indonesia. Glob Health Action. 2019;12: 1552652. doi:10.1080/16549716.2018.1552652

10. Souza RCSNP, Assunção RM, Oliveira DM, Neill DB, Meira W. Where did I get dengue? Detecting spatial clusters of infection risk with social network data. Spat Spatiotemporal Epidemiol. 2019;29: 163–175. doi:10.1016/j.sste.2018.11.005

11. Ramadona AL, Tozan Y, Lazuardi L, Rocklöv J. A combination of incidence data and mobility proxies from social media predicts the intra-urban spread of dengue in Yogyakarta, Indonesia. PLoS Negl Trop Dis. 2019;13: e0007298. doi:10.1371/journal.pntd.0007298

12. Davi C, Pastor A, Oliveira T, Neto FB de L, Braga-Neto U, Bigham AW, et al. Severe Dengue Prognosis Using Human Genome Data and Machine Learning. IEEE Trans Biomed Eng. 2019;66: 2861–2868. doi:10.1109/TBME.2019.2897285

13. Guo P, Zhang Q, Chen Y, Xiao J, He J, Zhang Y, et al. An ensemble forecast model of dengue in Guangzhou, China using climate and social media surveillance data. Sci Total Environ. 2019;647: 752–762. doi:10.1016/j.scitotenv.2018.08.044

14. Koh Y-M, Spindler R, Sandgren M, Jiang J. A model comparison algorithm for increased forecast accuracy of dengue fever incidence in Singapore and the auxiliary role of total precipitation information. Int J Environ Health Res. 2018;28: 535–552. doi:10.1080/09603123.2018.1496234

15. Carvajal TM, Viacrusis KM, Hernandez LFT, Ho HT, Amalin DM, Watanabe K. Machine learning methods reveal the temporal pattern of dengue incidence using meteorological factors in metropolitan Manila, Philippines. BMC Infect Dis. 2018;18: 183. doi:10.1186/s12879-018-3066-0

16. Baquero OS, Santana LMR, Chiaravalloti-Neto F. Dengue forecasting in São Paulo city with generalized additive models, artificial neural networks and seasonal autoregressive integrated moving average models. PLoS One. 2018;13: e0195065. doi:10.1371/journal.pone.0195065

17. Chen Y, Chu CW, Chen MIC, Cook AR. The utility of LASSO-based models for real time forecasts of endemic infectious diseases: A cross country comparison. J Biomed Inform. 2018;81: 16–30. doi:10.1016/j.jbi.2018.02.014

18. Villanes A, Griffiths E, Rappa M, Healey CG. Dengue Fever Surveillance in India Using Text Mining in Public Media. Am J Trop Med Hyg. 2018;98: 181–191. doi:10.4269/ajtmh.17-0253

19. Guo P, Liu T, Zhang Q, Wang L, Xiao J, Zhang Q, et al. Developing a dengue forecast model using machine learning: A case study in China. PLoS Negl Trop Dis. 2017;11: e0005973. doi:10.1371/journal.pntd.0005973

20. Chatterjee S, Dey N, Shi F, Ashour AS, Fong SJ, Sen S. Clinical application of modified bag-of-features coupled with hybrid neural-based classifier in dengue fever classification using gene expression data. Med Biol Eng Comput. 2018;56: 709–720. doi:10.1007/s11517-017-1722-y

21. Guo P, Wang L, Zhang Y, Luo G, Zhang Y, Deng C, et al. Can internet search queries be used for dengue fever surveillance in China? Int J Infect Dis. 2017;63: 74–76. doi:10.1016/j.ijid.2017.08.001

22. Yang S, Kou SC, Lu F, Brownstein JS, Brooke N, Santillana M. Advances in using Internet searches to track dengue. PLoS Comput Biol. 2017;13: e1005607. doi:10.1371/journal.pcbi.1005607

23. Marques-Toledo C de A, Degener CM, Vinhal L, Coelho G, Meira W, Codeço CT, et al. Dengue prediction by the web: Tweets are a useful tool for estimating and forecasting Dengue at country and city level. PLOS Neglected Tropical Diseases. 2017;11: e0005729. doi:10.1371/journal.pntd.0005729

24. Premaratne MK, Perera SSN, Malavige GN, Jayasinghe S. Mathematical Modelling of Immune Parameters in the Evolution of Severe Dengue. Comput Math Methods Med. 2017;2017: 2187390. doi:10.1155/2017/2187390

25. Jayasundara SDP, Perera SSN, Malavige GN, Jayasinghe S. Mathematical modelling and a systems science approach to describe the role of cytokines in the evolution of severe dengue. BMC Syst Biol. 2017;11: 34. doi:10.1186/s12918-017-0415-3

26. Li Z, Liu T, Zhu G, Lin H, Zhang Y, He J, et al. Dengue Baidu Search Index data can improve the prediction of local dengue epidemic: A case study in Guangzhou, China. PLoS Negl Trop Dis. 2017;11: e0005354. doi:10.1371/journal.pntd.0005354

27. Kesorn K, Ongruk P, Chompoosri J, Phumee A, Thavara U, Tawatsin A, et al. Morbidity Rate Prediction of Dengue Hemorrhagic Fever (DHF) Using the Support Vector Machine and the Aedes aegypti Infection Rate in Similar Climates and Geographical Areas. PLoS One. 2015;10: e0125049. doi:10.1371/journal.pone.0125049

28. Dayama P, Sampath K. Dengue disease outbreak detection. Stud Health Technol Inform. 2014;205: 1105–1109.

29. Sampath K, Dayama P. Predicting the operations alert levels for dengue surveillance and control. Stud Health Technol Inform. 2014;205: 1100–1104.

30. Gluskin RT, Johansson MA, Santillana M, Brownstein JS. Evaluation of Internet-based dengue query data: Google Dengue Trends. PLoS Negl Trop Dis. 2014;8: e2713. doi:10.1371/journal.pntd.0002713

31. Flamand C, Fabregue M, Bringay S, Ardillon V, Quénel P, Desenclos J-C, et al. Mining local climate data to assess spatiotemporal dengue fever epidemic patterns in French Guiana. J Am Med Inform Assoc. 2014;21: e232-240. doi:10.1136/amiajnl-2013-002348

32. Torres C, Barguil S, Melgarejo M, Olarte A. Fuzzy model identification of dengue epidemic in Colombia based on multiresolution analysis. Artif Intell Med. 2014;60: 41–51. doi:10.1016/j.artmed.2013.11.008

33. Buczak AL, Koshute PT, Babin SM, Feighner BH, Lewis SH. A data-driven epidemiological prediction method for dengue outbreaks using local and remote sensing data. BMC Med Inform Decis Mak. 2012;12: 124. doi:10.1186/1472-6947-12-124

34. Hoen AG, Keller M, Verma AD, Buckeridge DL, Brownstein JS. Electronic event-based surveillance for monitoring dengue, Latin America. Emerg Infect Dis. 2012;18: 1147–1150. doi:10.3201/eid1807.120055

35. Althouse BM, Ng YY, Cummings DAT. Prediction of dengue incidence using search query surveillance. PLoS Negl Trop Dis. 2011;5: e1258. doi:10.1371/journal.pntd.0001258

36. Chan EH, Sahai V, Conrad C, Brownstein JS. Using web search query data to monitor dengue epidemics: a new model for neglected tropical disease surveillance. PLoS Negl Trop Dis. 2011;5: e1206. doi:10.1371/journal.pntd.0001206

37. Faisal T, Taib MN, Ibrahim F. Neural network diagnostic system for dengue patients risk classification. J Med Syst. 2012;36: 661–676. doi:10.1007/s10916-010-9532-x

38. Ibrahim F, Faisal T, Salim MIM, Taib MN. Non-invasive diagnosis of risk in dengue patients using bioelectrical impedance analysis and artificial neural network. Med Biol Eng Comput. 2010;48: 1141–1148. doi:10.1007/s11517-010-0669-z

39. Syamsuddin M, Fakhruddin M, Sahetapy-Engel JTM, Soewono E. Causality Analysis of Google Trends and Dengue Incidence in Bandung, Indonesia With Linkage of Digital Data Modeling: Longitudinal Observational Study. J Med Internet Res. 2020;22: e17633. doi:10.2196/17633

40. Romero-Alvarez D, Parikh N, Osthus D, Martinez K, Generous N, Del Valle S, et al. Google Health Trends performance reflecting dengue incidence for the Brazilian states. BMC Infect Dis. 2020;20: 252. doi:10.1186/s12879-020-04957-0

41. Liu D, Guo S, Zou M, Chen C, Deng F, Xie Z, et al. A dengue fever predicting model based on Baidu search index data and climate data in South China. PLoS One. 2019;14: e0226841. doi:10.1371/journal.pone.0226841

42. Musa SS, Zhao S, Chan H-S, Jin Z, He DH. A mathematical model to study the 2014-2015 large-scale dengue epidemics in Kaohsiung and Tainan cities in Taiwan, China. Math Biosci Eng. 2019;16: 3841–3863. doi:10.3934/mbe.2019190

43. Messina JP, Brady OJ, Golding N, Kraemer MUG, Wint GRW, Ray SE, et al. The current and future global distribution and population at risk of dengue. Nat Microbiol. 2019;4: 1508–1515. doi:10.1038/s41564-019-0476-8

44. Titus Muurlink O, Stephenson P, Islam MZ, Taylor-Robinson AW. Long-term predictors of dengue outbreaks in Bangladesh: A data mining approach. Infect Dis Model. 2018;3: 322–330. doi:10.1016/j.idm.2018.11.004

45. Marques-Toledo CA, Bendati MM, Codeço CT, Teixeira MM. Probability of dengue transmission and propagation in a non-endemic temperate area: conceptual model and decision risk levels for early alert, prevention and control. Parasit Vectors. 2019;12: 38. doi:10.1186/s13071-018-3280-z

46. Verma M, Kishore K, Kumar M, Sondh AR, Aggarwal G, Kathirvel S. Google Search Trends Predicting Disease Outbreaks: An Analysis from India. Healthc Inform Res. 2018;24: 300–308. doi:10.4258/hir.2018.24.4.300

47. Ho CC, Ting C-Y, Raja DB. Using Public Open Data to Predict Dengue Epidemic: Assessment of Weather Variability, Population Density, and Land use as Predictor Variables for Dengue Outbreak Prediction using Support Vector Machine. Indian Journal of Science and Technology. 2018;11. doi:10.17485/ijst/2018/v11i4/115405

48. Phakhounthong K, Chaovalit P, Jittamala P, Blacksell SD, Carter MJ, Turner P, et al. Predicting the severity of dengue fever in children on admission based on clinical features and laboratory indicators: application of classification tree analysis. BMC Pediatr. 2018;18: 109. doi:10.1186/s12887-018-1078-y

49. Strauss RA, Castro JS, Reintjes R, Torres JR. Google dengue trends: An indicator of epidemic behavior. The Venezuelan Case. Int J Med Inform. 2017;104: 26–30. doi:10.1016/j.ijmedinf.2017.05.003

50. Nsoesie EO, Flor L, Hawkins J, Maharana A, Skotnes T, Marinho F, et al. Social Media as a Sentinel for Disease Surveillance: What Does Sociodemographic Status Have to Do with It? PLoS Curr. 2016;8. doi:10.1371/currents.outbreaks.cc09a42586e16dc7dd62813b7ee5d6b6

51. Liu K, Wang T, Yang Z, Huang X, Milinovich GJ, Lu Y, et al. Using Baidu Search Index to Predict Dengue Outbreak in China. Sci Rep. 2016;6: 38040. doi:10.1038/srep38040

52. Ximenes R, Amaku M, Lopez LF, Coutinho FAB, Burattini MN, Greenhalgh D, et al. The risk of dengue for non-immune foreign visitors to the 2016 summer olympic games in Rio de Janeiro, Brazil. BMC Infect Dis. 2016;16: 186. doi:10.1186/s12879-016-1517-z

53. Mohamad Mohsin MF, Abu Bakar A, Hamdan AR. Outbreak detection model based on danger theory. Appl Soft Comput. 2014;24: 612–622. doi:10.1016/j.asoc.2014.08.030

54. Puengpreeda A, Yhusumrarn S, Sirikulvadhana S. Weekly Forecasting Model for Dengue Hemorrhagic Fever Outbreak in Thailand. Eng J-Thail. 2020;24: 71–87. doi:10.4186/ej.2020.24.3.71

55. Amin S, Uddin MI, Hassan S, Khan A, Nasser N, Alharbi A, et al. Recurrent Neural Networks With TF-IDF Embedding Technique for Detection and Classification in Tweets of Dengue Disease. IEEE Access. 2020;8: 131522–131533. doi:10.1109/ACCESS.2020.3009058

56. Manogaran G, Lopez D, Chilamkurti N. In-Mapper combiner based MapReduce algorithm for processing of big climate data. Futur Gener Comp Syst. 2018;86: 433–445. doi:10.1016/j.future.2018.02.048

57. Agarwal N, Koti SR, Saran S, Kumar AS. Data mining techniques for predicting dengue outbreak in geospatial domain using weather parameters for New Delhi, India. Curr Sci. 2018;114: 2281–2291. doi:10.18520/cs/v114/i11/2281-2291

58. Manogaran G, Lopez D. A Gaussian process based big data processing framework in cluster computing environment. Cluster Comput. 2018;21: 189–204. doi:10.1007/s10586-017-0982-5

59. Jahangir I, Abdul-Basit, Hannan A, Javed S. Prediction of Dengue Disease Through Data Mining by Using Modified Apriori Algorithm. Proceedings of the 4th Acm International Conference of Computing for Engineering and Sciences (icces’2018). New York: Assoc Computing Machinery; 2018. doi:10.1145/3213187.3287612

60. Husin NA, Alharogi A, Mustapha N, Hamdan H, Husin UA. Early Self-Diagnosis of Dengue Symptoms Using Fuzzy and Data Mining Approach. In: Nifa F a. A, Lin CK, Hussain A, editors. Proceedings of the 3rd International Conference on Applied Science and Technology (icast’18). Melville: Amer Inst Physics; 2018. p. 020048. doi:10.1063/1.5055450

61. Anggraeni W, Pramudita G, Riksakomara E, Radityo PW, Samopa F, Pujiadi, et al. Artificial Neural Network for Health Data Forecasting, Case Study: Number of Dengue Hemorrhagic Fever Cases in Malang Regency, Indonesia. 2018 International Conference on Electrical Engineering and Computer Science (icecos). New York: Ieee; 2018. pp. 207–212.

62. Dennison Livelo E, Cheng C. Intelligent Dengue Infoveillance Using Gated Recurrent Neural Learning and Cross-Label Frequencies. 2018 Ieee International Conference on Agents (ica). New York: Ieee; 2018. pp. 2–7.

63. Wiratmadja II, Salamah SY, Govindaraju R. Healthcare Data Mining: Predicting Hospital Length of Stay of Dengue Patients. J Eng Technol Sci. 2018;50: 110–126. doi:10.5614/j.eng.technol.sci.2018.50.1.8

64. Arafiyah R, Hermin F. Data mining for dengue hemorrhagic fever (DHF) prediction with naive Bayes method. 1st International Conference of Education on Sciences, Technology, Engineering, and Mathematics (ice-Stem). Bristol: Iop Publishing Ltd; 2018. p. 012077. doi:10.1088/1742-6596/948/1/012077

65. Abuhamad HIS, Abu Bakar A, Zainudin S, Sahani M, Ali ZM. Feature Selection Algorithms for Malaysian Dengue Outbreak Detection Model. Sains Malays. 2017;46: 255–265. doi:10.17576/jsm-2017-4602-10

66. Manivannan P, Devi PI. Dengue Fever Prediction Using K-Means Clustering Algorithm. 2017 Ieee International Conference on Intelligent Techniques in Control, Optimization and Signal Processing (incos). New York: Ieee; 2017.

67. Dharmawardana KGS, Lokuge JN, Dassanayake PSB, Sirisena ML, Fernando ML, Perera AS, et al. Predictive Model for the Dengue Incidences in Sri Lanka Using Mobile Network Big Data. 2017 Ieee International Conference on Industrial and Information Systems (iciis). New York: Ieee; 2017. pp. 278–283.

68. Espina K, Estuar MRJE. Infodemiology for Syndromic Surveillance of Dengue and Typhoid Fever in the Philippines. In: CruzCunha MM, Varajao JEQ, Rijo R, Martinho R, Peppard J, SanCristobal JR, et al., editors. Centeris 2017 - International Conference on Enterprise Information Systems / Projman 2017 - International Conference on Project Management / Hcist 2017 - International Conference on Health and Social Care Information Systems and Technologies, Centeri. Amsterdam: Elsevier Science Bv; 2017. pp. 554–561. doi:10.1016/j.procs.2017.11.073

69. Rahim NF, Taib SM, Abidin AIZ. Dengue Fatality Prediction Using Data Mining. J Fundam Appl Sci. 2017;9: 671–683. doi:10.4314/jfas.v9i6s.52

70. Klein GH, Neto PG, Tezza R. Big Data and social media: surveillance of networks as management tool. Saude Soc. 2017;26: 208–217. doi:10.1590/S0104-12902017164943

71. Kerdprasop N, Kerdprasop K. Remote Sensing Based Modeling of Dengue Outbreak with Regression and Binning Classification. 2016 2nd Ieee International Conference on Computer and Communications (iccc). New York: Ieee; 2016. pp. 46–49.

72. Anggraeni W, Aristiani L. Using Google Trend Data in Forecasting Number of Dengue Fever Cases with ARIMAX Method Case Study : Surabaya, Indonesia. Proceedings of 2016 International Conference on Information & Communication Technology and Systems (icts). New York: Ieee; 2016. pp. 114–118.

73. Mathulamuthu SS, Asirvadam VS, Dass SC, Gill BS, Loshini T. Predicting Dengue Incidences Using Cluster Based Regression on Climate Data. 2016 6th Ieee International Conference on Control System, Computing and Engineering (iccsce). New York: Ieee; 2016. pp. 245–250.

74. Rahmawati D, Huang Y-P. Using C-support Vector Classification to Forecast Dengue Fever Epidemics in Taiwan. In: Wang WJ, Lee PJ, Er MJ, Jeng JT, editors. 2016 International Conference on System Science and Engineering (icsse). New York: Ieee; 2016.

75. Missier P, Romanovsky A, Miu T, Pal A, Daniilakis M, Garcia A, et al. Tracking Dengue Epidemics Using Twitter Content Classification and Topic Modelling. In: Casteleyn S, Dolog P, Pautasso C, editors. Current Trends in Web Engineering, Icwe 2016 International Workshops. Cham: Springer International Publishing Ag; 2016. pp. 80–92. doi:10.1007/978-3-319-46963-8_7

76. Abeyrathna MP a. R, Abeygunawrdane DA, Wijesundara R a. a. V, Mudalige VB, Bandara M, Perera S, et al. Dengue Propagation Prediction using Human Mobility. 2nd International Mercon 2016 Moratuwa Engineering Research Conference. New York: Ieee; 2016. pp. 156–161.

77. Fathima AS, Manimeglai D. Analysis of Significant Factors for Dengue Infection Prognosis Using the Random Forest Classifier. Int J Adv Comput Sci Appl. 2015;6: 240–245.

78. Tazkia RAK, Narita V, Nugroho AS. Dengue Outbreak Prediction for GIS based Early Warning System. 2015 International Conference on Science in Information Technology (ICSITech). New York: Ieee; 2015. pp. 121–125.

79. Wu Y, Lee G, Fu X, Hung T. Detect climatic factors contributing to dengue outbreak based on wavelet, support vector machines and genetic algorithm. In: Ao SI, Gelman L, Hukins DWL, Hunter A, Korsunsky AM, editors. World Congress on Engineering 2008, Vols I-Ii. Hong Kong: Int Assoc Engineers-Iaeng; 2008. pp. 303-+.

80. Salam N, Deeba F, Qadir F, Al-Hijli F, Al-Otaibi YN. Analysis of Correlation between Google Search Trends and Dengue Outbreaks from India. J Clin Diagn Res. 2019;13: LC13–LC15. doi:10.7860/JCDR/2019/42611.13304

81. Chire Saire JE. Building Intelligent Indicators to Detect Dengue Epidemics in Brazil using Social Networks. OrjuelaCanon AD, editor. 2019 Ieee Colombian Conference on Applications in Computational Intelligence (colcaci). New York: Ieee; 2019.

82. Swain S, Seeja KR. Analysis of Epidemic Outbreak in Delhi Using Social Media Data. In: Kaushik S, Gupta D, Kharb L, Chahal D, editors. Information, Communication and Computing Technology. Singapore: Springer-Verlag Singapore Pte Ltd; 2017. pp. 25–34.

83. Saravanan N, Gayathri V. Classification of Dengue Dataset Using J48 Algorithm and Ant Colony Based Aj48 Algorithm. New York: Ieee; 2017.

84. Carlos MA, Nogueira M, Machado RJ. Analysis of Dengue Outbreaks Using Big Data Analytics and Social Networks. 2017 4th International Conference on Systems and Informatics (icsai). New York: Ieee; 2017. pp. 1592–1597.

85. Ye X, Li S, Yang X, Qin C. Use of Social Media for the Detection and Analysis of Infectious Diseases in China. ISPRS Int Geo-Inf. 2016;5: 156. doi:10.3390/ijgi5090156

86. Li W, Chen Y. Risk Factor Identification and Spatiotemporal Diffusion Path During the Dengue Outbreak. In: Weng Q, Gamba P, Xian G, Chen JM, Liang S, editors. 2016 4rth International Workshop on Earth Observation and Remote Sensing Applications (EORSA). New York: Ieee; 2016.

87. Fathima S, Hundewale N. Comparison of Classification Techniques-SVM and Naives Bayes to predict the Arboviral Disease-Dengue. In: Chen B, Chen J, Chen X, Chen Y, Cho YR, Cui J, et al., editors. 2011 Ieee International Conference on Bioinformatics and Biomedicine Workshops. Los Alamitos: Ieee Computer Soc; 2011. pp. 538–539.

88. Srilekha G, Assistant Professor CD, Anupama B, Assistant Professor CD. Prediction of Dengue Outbreaks with Big Data using Machine Learning. GEDRAG &amp; ORGANISATIE REVIEW. 33. Available: https://www.academia.edu/42849518/Prediction_of_Dengue_Outbreaks_with_Big_Data_using_Machine_Learning

89. Ganthimathi M, Thangamani M, Mallika C, Prasanna Balaji V. Prediction of dengue fever using intelligent classifier. International Journal of Emerging Trends in Engineering Research. 2020;8: 1338–1341. doi:10.30534/ijeter/2020/65842020

90. Kumar NK, Sikamani KT. Prediction of chronic and infectious diseases using machine learning classifiers-A systematic approach. International Journal of Intelligent Engineering and Systems. 2020;13: 11–20. doi:10.22266/IJIES2020.0831.02

91. Guiyab RB. Development of prediction models for the dengue survivability prediction: An integration of data mining and decision support system. International Journal of Innovative Technology and Exploring Engineering. 2019;8: 2199–2205. doi:10.35940/ijitee.J9411.0881019

92. Chovatiya M, Dhameliya A, Deokar J, Gonsalves J, Mathur A. Prediction of dengue using recurrent neural network. 2019. pp. 926–929. doi:10.1109/icoei.2019.8862581

93. Kerdprasop K, Kerdprasop N, Chansilp K, Chuaybamroong P. The Use of Spaceborne and Oceanic Sensors to Model Dengue Incidence in the Outbreak Surveillance System. Lecture Notes in Computer Science (including subseries Lecture Notes in Artificial Intelligence and Lecture Notes in Bioinformatics). 2019;11619 LNCS: 447–460. doi:10.1007/978-3-030-24289-3_33

94. Link H, Richter SN, Leung VJ, Brost RC, Phillips CA, Staid A. Statistical models of dengue fever. Communications in Computer and Information Science. 2019;996: 175–186. doi:10.1007/978-981-13-6661-1_14

95. Arafiyah R, Hermin F, Kartika IR, Alimuddin A, Saraswati I. Classification of Dengue Haemorrhagic Fever (DHF) using SVM, naive bayes and random forest. 2018. doi:10.1088/1757-899X/434/1/012070

96. Mishra S, Tripathy HK, Panda AR. An improved and adaptive attribute selection technique to optimize Dengue fever prediction. International Journal of Engineering and Technology(UAE). 2018;7: 480–486. doi:10.14419/ijet.v7i2.29.13802

97. Wu C-H, Kao S-C, Kan M-H. Knowledge discovery in open data of dengue epidemic. 2017. doi:10.1145/3092090.3092093

98. Albinati J, Meira W Jr, Pappa GL, Teixeira M, Marques-Toledo C. Enhancement of epidemiological models for dengue fever based on twiter data. 2017. pp. 109–118. doi:10.1145/3079452.3079464

99. Zhu G, Hunter J, Jiang Y. Improved Prediction of Dengue Outbreak Using the Delay Permutation Entropy. 2017. pp. 828–832. doi:10.1109/iThings-GreenCom-CPSCom-SmartData.2016.172

100. Zainudin Z, Shamsuddin SM. Predictive analytics in Malaysian dengue data from 2010 until 2015 using BigML. International Journal of Advances in Soft Computing and its Applications. 2016;8: 18–30.

101. Milinovich GJ, Avril SMR, Clements ACA, Brownstein JS, Tong S, Hu W. Using internet search queries for infectious disease surveillance: Screening diseases for suitability. BMC Infectious Diseases. 2014;14. doi:10.1186/s12879-014-0690-1

102. Ongruk P, Siriyasatien P, Kesorn K. New key factors discovery to enhance dengue fever forecasting model. Advanced Materials Research. 2014;931–932: 1457–1461. doi:10.4028/www.scientific.net/AMR.931-932.1457

103. Balasundaram A, Bhuvaneswari PTV. Comparative study on decision tree based data mining algorithm to assess risk of epidemic. 2013. pp. 390–396. doi:10.1049/ic.2013.0344

104. Wu Y, Lee G, Fu X, Soh H, Hung T. Mining weather information in dengue outbreak: Predicting future cases based on wavelet, SVM and GA. Lecture Notes in Electrical Engineering. 2009;39 LNEE: 483–494. doi:10.1007/978-90-481-2311-7_41

105. Zhang Y, Ibaraki M, Schwartz FW. Disease surveillance using online news: Dengue and zika in tropical countries. Journal of Biomedical Informatics. 2020;102. doi:10.1016/j.jbi.2020.103374

106. Souza RCSNP. Detecting spatial clusters of infection risk with geo-located social media data. 2018.

107. Coberly JS, Fink CR, Elbert Y, Yoon I-K, Velasco JM, Tomayao AD, et al. Tweeting Fever: Can Twitter Be Used to Monitor the Incidence of Dengue-Like Illness in the Philippines? JOHNS HOPKINS APL TECHNICAL DIGEST. 2014;32: 12.

108. Gomide J, Veloso A, Meira Jr. W, Almeida V, Benevenuto F, Ferraz F, et al. Dengue surveillance based on a computational model of spatio-temporal locality of Twitter. 2011. doi:10.1145/2527031.2527049

109. Fang Z-H, Tzeng J-S, Chen CC, Chou T-C. A study of machine learning models in epidemic surveillance: Using the query logs of search engines. 2010. pp. 1438–1449.

110. Souza J, Leung CK, Cuzzocrea A. An Innovative Big Data Predictive Analytics Framework over Hybrid Big Data Sources with an Application for Disease Analytics. Advances in Intelligent Systems and Computing. 2020;1151 AISC: 669–680. doi:10.1007/978-3-030-44041-1_59

111. Yogapriya P, Geetha P. Dengue disease detection using K-means, hierarchical, kohonen-SOM clustering. International Journal of Innovative Technology and Exploring Engineering. 2019;8: 904–907. doi:10.35940/ijitee.J9066.0881019

112. Adias Sabara M, Somantri O, Nurcahyo H, Kurnia Achmadi N, Latifah U, Harsono. Diagnosis classification of dengue fever based on Neural Networks and Genetic algorithms. 2019. doi:10.1088/1742-6596/1175/1/012065

113. Jongmuenwai B, Lowanichchai S, Jabjone S. Comparision using data mining algorithm techniques for predicting of dengue fever data in northeastern of Thailand. 2019. pp. 532–535. doi:10.1109/ECTICon.2018.8619953

114. Balasaravanan K, Prakash M. Detection of dengue disease using artificial neural network based classification techniquetion. International Journal of Engineering and Technology(UAE). 2018;7: 13–15. doi:10.14419/ijet.v7i1.3.8978

115. Acosta Torres J, Oller Meneses L, Sokol N, Balado Sardiñas R, Montero Díaz D, Balado Sansón R, et al. Decision tree technique applied to the clinical method in the dengue diagnosis. Revista Cubana de Pediatria. 2016;88: 441–453.

116. Soonthornphisaj N, Thitiprayoonwongse D. Knowledge discovery on dengue patients using data mining techniques. 2016. pp. 371–375.

117. Fathima SA, Hundewale N. Comparitive analysis of machine learning techniques for classification of arbovirus. 2012. pp. 376–379. doi:10.1109/BHI.2012.6211593

118. Fathima S, Hundewale N. Comparison of classification techniques-SVM and naives bayes to predict the Arboviral disease-Dengue. 2011. pp. 538–539. doi:10.1109/BIBMW.2011.6112426

119. Long ZA, Abu Bakar A, Razak Hamdan A, Sahani M. Multiple attribute frequent mining-based for dengue outbreak. Lecture Notes in Computer Science (including subseries Lecture Notes in Artificial Intelligence and Lecture Notes in Bioinformatics). 2010;6440 LNAI: 489–496. doi:10.1007/978-3-642-17316-5_46
