# Supplementary material for: Data-driven methods for dengue prediction and surveillance using real-world and Big Data: A systematic review
Source: PLoS Negl Trop Dis. 2022 Jan 7;16(1):e0010056. doi: 10.1371/journal.pntd.0010056 (PMC8740963; doi:10.1371/journal.pntd.0010056)
Supplement: S4 Table — (DOCX) [file pntd.0010056.s008.docx]

**S4 Table. Studied outcomes for dengue surveillance and prediction**

| **Outcome** | **n** | **%** |
| --- | --- | --- |
| Dengue incidence rate | 58 | 49 |
| Dengue diagnosis | 20 | 17 |
| Dengue outbreak | 18 | 15 |
| Dengue severity | 8 | 7 |
| Dengue-related tweet | 4 | 3 |
|  |  |  |
| Other outcome | 18 | 15 |
| Dengue risk level | 3 | 2.5 |
| Dengue spatial cluster | 2 | 1.7 |
| Dengue mortality | 2 | 1.7 |
| Dengue transmission risk | 2 | 1.7 |
| Dengue serotype | 1 | 0.8 |
| Dengue maps | 1 | 0.8 |
| Dynamic mobility-weighted incidence index | 1 | 0.8 |
| Length of hospital stay | 1 | 0.8 |
| Peak time of dengue incidence | 1 | 0.8 |
| Peak value | 1 | 0.8 |
| R0 | 1 | 0.8 |
| Trend in dengue articles | 1 | 0.8 |
| Yearly dengue deaths | 1 | 0.8 |
